# Immunoprofiles of human Sertoli cells infected with Zika virus reveals unique insights into host-pathogen crosstalk

**DOI:** 10.1038/s41598-018-27027-7

**Published:** 2018-06-07

**Authors:** Daniel P. Strange, Richard Green, David N. Siemann, Michael Gale, Saguna Verma

**Affiliations:** 1 Department of Tropical Medicine, Medical Microbiology and Pharmacology, John A. Burns School of Medicine, University of Hawaii at Manoa, Honolulu, Hawaii USA; 2 Department of Immunology, Center for Innate Immunity and Immune Disease, University of Washington School of Medicine, Seattle, Washington USA

## Abstract

Confirmed reports of Zika virus (ZIKV) in seminal fluid months after clearance of viremia suggests that ZIKV can establish persistent infection in the seminiferous tubules, an immune privileged site of the testis. The seminiferous tubule epithelium is mainly composed of Sertoli cells that function to nourish and protect developing germ cells. We recently demonstrated that primary human Sertoli cells (hSeC) were highly susceptible to ZIKV as compared to dengue virus without causing cell death and thus may act as a reservoir for ZIKV in the testes. However, the cellular and immune responses of hSeC to infection with ZIKV or any other virus are not yet characterized. Using genome-wide RNA-seq to compare immunoprofiles of hSeC, we show that the most prominent response to ZIKV at early stage of infection was suppression of cell growth and proliferation functional pathways. Peak virus replication was associated with induction of multiple antiviral defense pathways. Unique ZIKV-associated signatures included dysregulation of germ cell-Sertoli cell junction signaling. This study demonstrates that hSeC are capable of signaling through canonical pro-inflammatory pathways and provides insights into unique cell-type-specific response induced by ZIKV in association with viral persistence in the testes.

## Introduction

Zika virus (ZIKV) is an emerging mosquito-borne flavivirus that has quickly become a major public health concern. ZIKV remained in obscurity until the 2007 outbreak in the Western Pacific of Yap, followed by multiple smaller outbreaks in Pacific islands. These outbreaks led to the larger and current emerging epidemic in the Americas, displaying more severe disease outcomes including congenital brain abnormalities as early as 2015. In the U.S. alone, including U.S. territories, 42,688 cases of ZIKV infection have been reported to the CDC as of December 6, 2017. Of the cases reported in U.S. states, most were returning travelers from affected areas, but of the 276 cases acquired locally, 226 were mosquito-borne and 51 were acquired through other routes, including sexual transmission^1^. Recent reports of ZIKV detection in human semen and spermatozoa, as well as confirmed cases of ZIKV sexual transmission, also distinguishes ZIKV from other closely related flaviviruses in terms of transmissibility^2,3^. Evidence shows that ZIKV can be spread sexually by asymptomatic, symptomatic, and post-viremic males^4^. Further, a recent study reported that 56% of ZIKV serum-positive males were also semen-positive for ZIKV RNA up to 108 days after symptoms onset^5^, suggesting a much longer infectious phase of ZIKV as compared to other mosquito-borne flaviviruses.

Detection of ZIKV in the seminal fluid and spermatozoa for months after viremia has cleared^2,3,5^ provides indirect evidence that ZIKV establishes persistent infection within seminiferous tubules, an immune privileged compartment of the testis. However, the key pathogenic features leading to this persistence, such as the route of ZIKV entry and mechanisms of host evasion remains obscure. Recent animal model studies have demonstrated that ZIKV infects mouse Leydig cells, Sertoli cells, and spermatagonia, resulting in damaged testicular tissue and reduction in motile sperm^6,7^. However, due to the immune-deficient nature of mouse models, these studies are limited in human predictive capacity, and thus, the important features of ZIKV infection in human testes, such as the specific effects on host immune response, remain poorly defined.

The mammalian testis is composed of two main compartments, the interstitial space and the seminiferous tubules^8,9^. The interstitial space contains blood vessels, immune cells, and testosterone-producing Leydig cells, whereas seminiferous tubules consist of peritubular cells, Sertoli cells (SC), and developing germ cells^8,9^. SC are large columnar cells that form the so-called blood-testis barrier (BTB), extending from the basal lamina of the seminiferous tubules into the lumen of the tubular compartment and function as nurse cells to developing germ cells as they mature to spermatozoa during spermatogenesis^8,9^. *In vivo*, mammalian SC cease to divide post-puberty, which is a characteristic that likely serves to maintain the structural integrity of the BTB^10^. *In vitro*, primary human SC are shown to divide very slowly, approximately every 4 days^11^, and only up to passage 8 or 9. The immune privileged nature of the testis is required to protect delicate post-meiotic germ cells from systemic immune attack and pro-inflammatory milieu caused by infection. In rodents, SC are shown to be inherently immunosuppressive, constitutively expressing high levels of anti-inflammatory molecules such as transforming growth factor (TGF) β1–β3 and activin A^9^. Moreover, *in vitro* studies have shown that SC can also elicit innate immune responses upon stimulation with various TLR agonists such as LPS, flagellin, and peptidoglycan^12,13^, indicating a dichotomous role of SC in directing the testicular immune response. However, the specific innate immune response elicited by human SC to ZIKV or any testes-tropic virus is yet to be characterized. Further, Data regarding global immune response to any pathogen in both mouse and human SC is lacking, thus limiting our understanding of the specific immune mechanisms associated with virus persistence in the immune privilege compartment of the testes, including how they affect germ cell survival.

We have recently shown that primary human SC can support ZIKV infection with higher efficiency as compared to dengue virus (DENV) without any observable cytopathic effects^14^. We further demonstrated that ZIKV can efficiently cross the *in vitro* blood-testis barrier and migrate to the luminal side of the barrier^14^. Together, these observations indicate that SC may act as a reservoir for long-term replication of virus in the testes, therefore allowing ZIKV to continually infect germ cells and developing spermatocytes even after peripheral clearance. Considering the crucial role of SC in sperm development and in maintaining immune homeostasis of the seminiferous tubular compartment, we believe that investigating the global gene changes modulated by ZIKV in human SC will provide important insights into the unique molecular events that occur in human testicular tissue following infection. The present study utilizes RNA-sequencing (RNA-seq) technology as an unbiased approach to profile the host transcriptome and analyze the temporal effects of ZIKV infection on primary human SC (hSeC). Our results indicate that ZIKV induced pro-inflammatory mediators and genes associated with various innate immune response pathways. Moreover, cell growth and proliferation pathways were dysregulated by ZIKV infection in these cells. Unique ZIKV-associated response in hSeC included dysregulation of genes involved in the germ cell-Sertoli cell junction signaling pathway. Collectively, the data presented here provides novel insights into the innate immune response and cell signaling pathways associated with ZIKV infection of human SC and offers a unique molecular framework for future research to understand ZIKV persistence in human testes.

## Results

### Distinct genes and pathways in hSeC were temporally modulated by ZIKV infection

**Figure 1:**
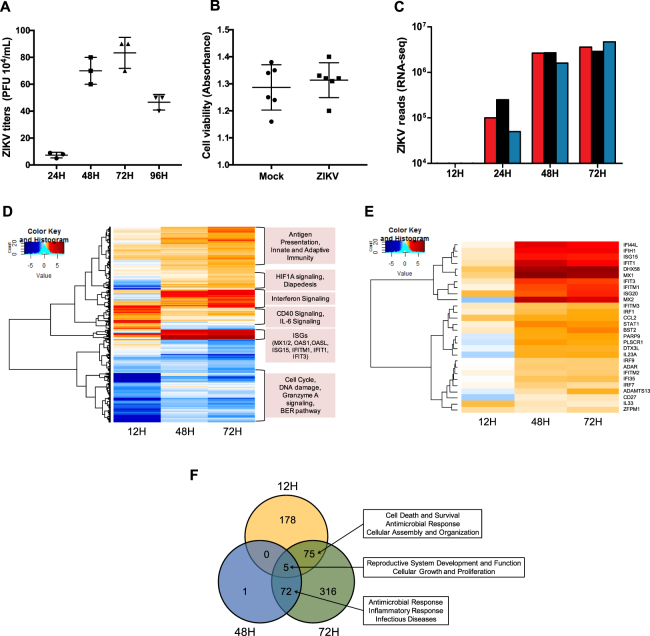
RNA-seq analysis reveals differentially expressed genes in ZIKV-infected hSeC. (**A**) ZIKV titers in supernatant measured using plaque assay (n = 3 per time point). (**B**) ZIKV did not induce cytopathic effect in hSeC at 72 hpi as compared to mock. Cytopathic effect assessed using the CellTiter 96 AQueous One Solution cell proliferation assay kit, and percent cell viability was calculated by comparison to mock-infected cells. (**C**) ZIKV genome reads from RNA-seq analysis (n = 3 per time point). (**D,E**) Differentially expressed genes (DEGs) across all time points (log2 FC >|1.5|, p < 0.05) determined using voom and limma packages in Bioconductor. (**D**) Global heat map of DEGs shows downregulation of cell cycle, DNA damage, granzyme A signaling, and BER pathways at 12 hpi, and upregulation of interferon signaling, including ISGs at 48 and 72 hpi. (**E**) Heat map of interferon signaling. Heat maps were generated using gplots and WGCNA Bioconductor packages. (**F**) Venn diagram of DEGs for each time point by significance alone (p < 0.05) identifying top modulated functional networks between time points (determined by IPA).

hSeC were infected with ZIKV strain PRVABC59 at the multiplicity of infection (MOI) of 1 and the virus was detected by plaque assay at 24 hrs post infection (hpi), with peak virus titers of 6 log10 PFU/mL observed at 72 hpi (Fig. 1A). To investigate the effect of ZIKV infection on hSeC survival, we also measured hSeC viability at 72 hpi. As shown in Fig. 1B, cell viability of infected hSeC was more than 97% in comparison to mock, indicating that peak virus replication had no significant cytopathic effect. Based on these initial results, 12, 24, 48, and 72 hpi were selected for RNA-seq analysis to determine the temporal transcriptome changes in hSeC following ZIKV infection. Consistent with the plaque results (Fig. 1A), initial RNA-seq analysis showed similar trend of increasing ZIKV reads over the course of infection (Fig. 1C). Together, these results show that ZIKV can maintain a productive infection in hSeC and are consistent with our recent study demonstrating that ZIKV does not compromise hSeC viability for up to 9 days after infection^14^.

Subsequent transcriptome profiling and comparison of mock-infected samples with ZIKV-infected samples revealed a total of 647 non-redundant differentially expressed genes (DEGs) across observations (p < 0.05; Supplementary Tables S1–S3). Since hSeC divide very slowly *in vitro* (~4 days)^11^, ZIKV-infected hSeC for all time points were compared to 24-hour mock controls. Although viral reads were observed at 24 hpi (Fig. 1C), the gene expression changes at this time point were not significant in comparison to mock (p < 0.05) and therefore were removed from the remaining analyses. Global heat map analysis of DEGs inclusive of a log2 FC cutoff of >|1.5| indicated that at 12 hpi, most were suppressed or “downregulated”, in contrast to 48 and 72 hpi, where majority were induced or “upregulated” (Fig. 1D). The downregulated genes, mainly at 12 hpi, were involved in cell cycle, DNA damage and repair, and granzyme A signaling (Fig. 1D). Whereas most of the upregulated genes at later time points, during peak virus replication, were implicated in innate immune response and interferon (IFN) signaling (Fig. 1D). Further heat map analysis of IFN signaling (Fig. 1E) provides a closer depiction of upregulated IFN stimulated genes (ISGs) at 48 and 72 hpi. These ISGs included genes known to be responsive directly to interferon regulatory factor (IRF)3 activation and signaling, including IFN induced protein with tetratricopeptide repeats 1 and 3 (*IFIT1* and *IFIT3*) and IFN stimulated gene 15 (*ISG15*)^15^, and genes only induced through IFN, including IFN induced protein 44 like (*IFI44L*), IFN stimulated exonuclease gene 20 (*ISG20*), IFN induced transmembrane protein 1 (*IFITM1*), DExH-box helicase 58 (*DHX58*), IFN induced helicase C domain 1 (*IFIH1*), and MX dynamin-like GTPase 1 and 2 (*MX1* and *MX2*) genes. In general, the host IFN response, through activation of IRF3, IFN production, and induction of ISGs, is crucial for mounting the innate antiviral defense mechanisms that block virus replication and maturation at different levels of infection^16–18^. Therefore, the upregulation of various IRF3-target genes and ISGs observed at 48 and 72 hpi indicates that hSeC can initiate and mount a robust antiviral response during peak ZIKV replication. DEGs at 12, 48, and 72 hpi (Supplementary Tables S1–S3) based on significance alone (p < 0.05) were further processed for pathway enrichment using Ingenuity Pathway Analysis (IPA) by Qiagen to identify the top functional networks temporally modulated in hSeC following ZIKV infection. Of these DEGs, 178 were differentially expressed exclusively at 12 hpi and 316 exclusively at 72 hpi (Fig. 1F).

### Early ZIKV infection of hSeC induced a biased suppression of cellular growth and proliferation pathways

**Figure 2:**
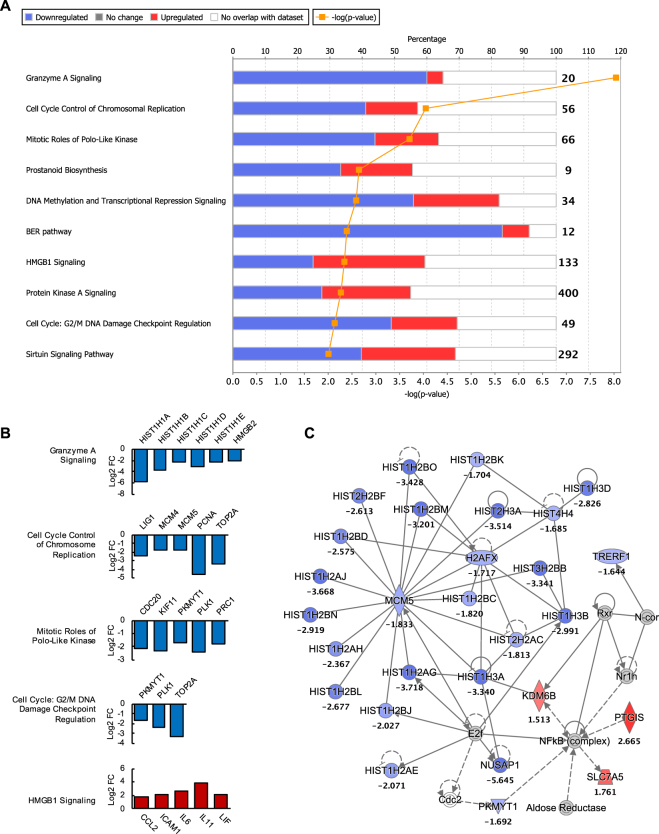
Biological pathways and functional networks modulated by ZIKV in hSeC at 12 hpi. (**A**) Top IPA Canonical Pathways across entire dataset at 12 hpi. Pathways order by most significant (Fisher’s exact test right-tailed). Cellular growth and proliferation (cell cycle, DNA replication and repair) pathways were predominantly downregulated at 12 hpi. Stacked bars represent percentage of genes modulated and/or IPA predicted out of total (in bold, right) in pathway. (**B**) Significant DEGs (p < 0.05; determined by limma package in Bioconductor) of select cellular growth and proliferation pathways and HMGB1 Signaling pathway at 12 hpi meeting log2 FC cutoff of >|1.5|. (**C**) Top scoring IPA functional network at 12 hpi: Cellular Assembly and Organization, DNA Replication, Recombination, and Repair, Post-Translational Modification network. Solid line between nodes represents direct interaction and dashed line represents indirect interaction. Nodes ranked by log2 FC. Node shapes represent functional classes of gene products. Vertical diamond shaped nodes represent enzymes; horizontal diamond peptidases; trapezoid transporters; triangle kinases; horizontal oval transcription regulators; vertical oval transmembrane receptors; rectangle growth factors; circle proteins. Node color indicates level of expression. Red is upregulated, and blue is downregulated. IPA functional network cutoff criteria for DEGs was log2 FC >|1.5| (p < 0.05).

DEGs at 12 hpi (Supplementary Table S1) enriched via IPA (log2 FC cutoff of >|1.5|) revealed suppression of pathways involved in cellular growth and proliferation. Majority of these downregulated pathways included those involved in cell cycle regulation (Cell Cycle Control and Chromosomal Replication; Mitotic Roles of Polo-Like Kinase; DNA Methylation and Transcriptional Repression; Cell Cycle: G2/M DNA Damage Checkpoint Regulation; GADD45 Signaling) and cellular stress and injury (BER pathway; HMGB1 Signaling) (Fig. 2A). Other top canonical pathways that were disproportionally downregulated at 12 hpi included Granzyme A Signaling, where almost 60% of the genes were downregulated (Fig. 2A). As shown in Fig. 2B, there were multiple genes that overlapped with multiple cellular growth pathways.

The top downregulated signatures at 12 hpi were predominately histone cluster 1 (HIST1) genes (Supplementary Table S1). IPA identified these genes to be associated with the functional network of Cellular Assembly and Organization, DNA Replication, Recombination, and Repair, and Post-Translational Modification (Fig. 2C). HIST1 cluster proteins, which are essential for nucleosome and chromatin formation, are downregulated in response to DNA damage involving functional p53 as well as inhibition of cyclin dependent kinases (CDKs)^19^. Consistent with this, IPA identified CDK inhibitor 1A (CDKN1A) and tumor protein p53 (TP53) among the top activated upstream regulators at 12 hpi (Table 1). CDKN1A functions by inhibiting the activity of CDK complexes, and thus serves as a regulator of cell cycle progression at various phases^20^. Whereas TP53 can directly activate DNA repair proteins in response to DNA damage and can stall the cell cycle at the G_1_/S regulation point upon DNA damage recognition^21,22^. The predicted activation of CDK1A and TP53 as well as the downregulation of HIST1 gene clusters may suggest a state of cell cycle arrest or cytostasis in hSeC at 12 hpi, which is consistent with the biased suppression of cellular growth and proliferation pathways observed at this stage of infection. Notably, upstream IPA analysis at 12 hpi also predicted significant activation of TGF-β1 (*TGFB1*) (Table 1), which is known to contribute to the immunosuppressive milieu of the testes^9^, thus indicating that ZIKV infection of hSeC may also affect important regulators of testicular immune privilege.

**Table 1:** Top 30 upstream regulators with predicted activity at 12 hpi by significance (Fisher’s exact test right-tailed).

Upstream Regulator	Expr Log Ratio	Molecule Type	Predicted Activation State	Activation z-score	p-value of overlap
CDKN1A	0.379	kinase	Activated	2.185	1.13E-25
TP53	−0.66	transcription regulator	Activated	2.877	2.96E-18
HGF		growth factor	Inhibited	−2.281	1.10E-16
PTGER2		g-protein coupled receptor	Inhibited	−2.013	4.46E-16
NUPR1	0.941	transcription regulator	Activated	5.292	1.76E-15
FOXM1	−1.99	transcription regulator	Inhibited	−2.915	3.02E-15
CSF2	1.284	cytokine	Inhibited	−2.295	1.01E-14
E2F3	0.212	transcription regulator	Inhibited	−2.515	1.93E-13
EP400	−0.265	other	Inhibited	−2.621	6.90E-12
IL6	2.707	cytokine	Activated	2.248	3.70E-11
RB1	−0.298	transcription regulator	Activated	2.595	4.24E-11
mir-21		microrna	Activated	3.614	5.25E-11
RABL6	0.015	other	Inhibited	−3.162	1.50E-10
F10		peptidase	Activated	2.041	1.75E-10
EDN1	1.025	cytokine	Activated	2.515	1.14E-09
TBX2	−0.171	transcription regulator	Inhibited	−3	1.39E-08
F2		peptidase	Activated	2.614	4.25E-08
P38 MAPK		group	Activated	2.342	6.92E-08
AGER	0.824	transmembrane receptor	Activated	2.495	1.07E-07
F2R	0.115	g-protein coupled receptor	Activated	2.454	1.10E-07
E2F1	−1.381	transcription regulator	Inhibited	−2.007	1.35E-07
HDAC2	−0.761	transcription regulator	Activated	2.449	1.40E-07
S1PR2	0.168	g-protein coupled receptor	Activated	2.197	1.54E-07
IL17A		cytokine	Activated	2.59	1.58E-07
TGFB1	0.052	growth factor	Activated	3.567	1.76E-07
OSM		cytokine	Activated	2.708	5.28E-07
F2RL1	−0.405	g-protein coupled receptor	Activated	2.586	6.58E-07
LEP		growth factor	Activated	2.476	6.97E-07
MITF		transcription regulator	Inhibited	−3.464	7.49E-07
HDAC1	−0.842	transcription regulator	Activated	2.135	8.55E-07

### Peak ZIKV replication induced a robust antiviral state by activating multiple pathogen recognition receptor signaling pathways in hSeC

**Figure 3:**
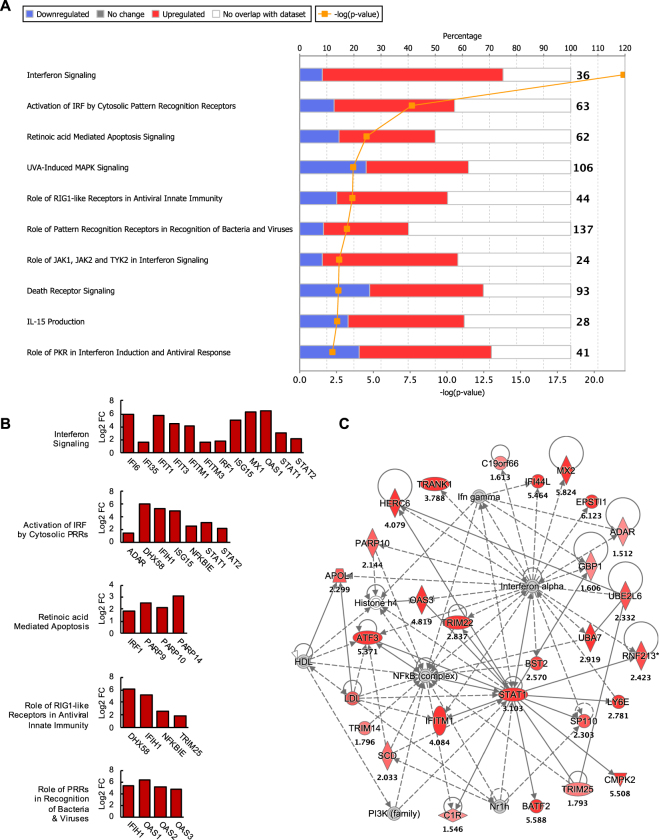
Biological pathways and functional networks modulated by ZIKV in hSeC at 48 hpi. (**A**) Top IPA Canonical Pathways across entire dataset at 48 hpi. Pathways order by most significant (Fisher’s exact test right-tailed). Antiviral and innate immune response pathways were predominantly upregulated. Stacked bars represent percentage of genes modulated and/or IPA predicted out of total (in bold, right) in pathway. (**B**) Significant DEGs (p < 0.05; determined by limma in Bioconductor) of select antiviral pathways at 48 hpi meeting log2 FC cutoff of >|1.5|. (**C**) Top scoring IPA functional network at 48 hpi: Infectious Diseases, Cell Signaling, Antimicrobial Response network. Solid line between nodes represents direct interaction and dashed line represents indirect interaction. Nodes ranked by log2 FC. Node shapes represent functional classes of gene products. Vertical diamond shaped nodes represent enzymes; horizontal diamond peptidases; trapezoid transporters; triangle kinases; horizontal oval transcription regulators; vertical oval transmembrane receptors; rectangle growth factors; circle proteins. Node color indicates level of expression. Red is upregulated, and blue is downregulated. IPA functional network cutoff criteria for DEGs was log2 FC >|1.5| (p < 0.05).

The peak ZIKV titers observed at 48 and 72 hpi were shown to induce gene expression of various cytokines, IRF3-target genes, and ISGs as determined by heat map analysis (Fig. 1D,E). Consistent with these preliminary results, DEGs at 48 hpi (Supplementary Table S2) enriched via IPA (log2 FC cutoff of >|1.5|) revealed that the top canonical pathways modulated at this stage of infection were predominately involved in innate immune response (Fig. 3A). These included key antiviral pathways such as Interferon Signaling, Activation of IRF by Cytosolic Pattern Recognition Receptors (PRRs), Role of RIG1-like Receptors in Antiviral Innate Immunity, and Role of PRRs in Recognition of Bacteria and Viruses (Fig. 3A). Among these, IFN signaling was the most significantly modulated pathway, which included significant upregulation of the ISGs *IFI6, IFI35, IFITM1, IFITM3, IRF1, MX1*, signal transducer and activator of transcription 1 (*STAT1*), and *STAT2*, as well as ISGs targeted directly by IRF3 such as *IFIT1*, *IFIT3, ISG15*, and *OAS1*^15^ (Fig. 3B). Other upregulated antiviral signatures noted in Fig. 3B included those involved in cytoplasmic recognition of RNA viruses, such as *DHX58* (LGP2), *IFIH1* (MDA5), and tripartite motif containing 25 (*TRIM25*). LGP2 and MDA5 are cytosolic viral RNA sensors, belonging to the retinoic acid-inducible gene I (RIG-I)-like receptor (RLR) family, that together, mediate the production of type I IFNs, antiviral effector genes, and pro-inflammatory cytokines^23,24^. Furthermore, also a top scoring pathway at 48 hpi, Retinoic acid Mediated Apoptosis, was characterized by the upregulation of *IRF1* and poly(ADP-ribose) polymerase (PARP) genes *PARP9*, *PARP10*, and *PARP14* (Fig. 3B), which also accounted for all upregulated genes in the Death Receptor Signaling pathway (Fig. 3A). PARPs are reported to be ISGs encoding for proteins that inhibit RNA virus replication and are thereby considered ISGs^25^. Based on these expression profiles, the top scoring IPA functional network modulated at 48 hpi was that of Infectious Diseases, Cell Signaling, and Antimicrobial Response (Fig. 3C). In addition, based on the downstream DEGs in the dataset, IPA predicted the activation of key RNA-sensing PRRs and IFN molecules, including RIG-I (*DDX58*), TLR3, TLR7, and type I (*IFNA1*, *IFNA2*, and *IFNB1*) and type III (*IFNL1*) IFNs (Table 2), all of which are implicated in innate immune response against RNA viruses^26–28^. Together, the antiviral genes and pathways induced at 48 hpi indicate that hSeC can mount a robust response against ZIKV through a variety of innate antiviral mechanisms.

**Table 2:** Top 30 upstream regulators with predicted activity at 48 hpi by significance (Fisher’s exact test right-tailed).

Upstream Regulator	Expr Log Ratio	Molecule Type	Predicted Activation State	Activation z-score	p-value of overlap
IFNL1		cytokine	Activated	5.226	2.83E-54
IFNA2		cytokine	Activated	5.709	6.75E-53
Interferon alpha		group	Activated	5.019	1.96E-52
PRL		cytokine	Activated	5.824	2.26E-48
IRF7	1.338	transcription regulator	Activated	5.168	3.98E-43
MAPK1	0.262	kinase	Inhibited	−5.196	4.25E-41
STAT1	3.103	transcription regulator	Activated	4.807	1.39E-37
CNOT7	−0.046	transcription regulator	Inhibited	−2.425	2.10E-36
IFNG		cytokine	Activated	6.157	2.93E-33
IL1RN		cytokine	Inhibited	−4	5.28E-32
IRF1	1.893	transcription regulator	Activated	4.149	1.11E-29
IRF3	−0.04	transcription regulator	Activated	4.6	1.50E-29
IFN Beta		group	Activated	4.357	2.27E-29
EIF2AK2	1.072	kinase	Activated	4.052	2.26E-27
NKX2-3		transcription regulator	Inhibited	−4.583	9.59E-27
IFNA1/IFNA13	−0.047	cytokine	Activated	3.794	2.48E-26
TRIM24	0.432	transcription regulator	Inhibited	−4.054	2.61E-26
TGM2	0.712	enzyme	Activated	4.439	9.61E-25
IRF5		transcription regulator	Activated	3.815	1.07E-24
Ifnar		group	Activated	3.843	4.52E-23
IFNB1		cytokine	Activated	4.027	8.17E-23
STAT2	2.166	transcription regulator	Activated	2.208	1.37E-22
MAVS	0.372	other	Activated	3.441	8.96E-22
Ifn		group	Activated	3.392	5.53E-21
TLR3		transmembrane receptor	Activated	3.384	1.65E-20
ACKR2		g-protein coupled receptor	Inhibited	−3.317	4.40E-20
IFNAR1	0.31	transmembrane receptor	Activated	2.213	7.80E-20
TLR7		transmembrane receptor	Activated	3.664	1.65E-19
IFNAR2	0.676	transmembrane receptor	Activated	2.236	3.88E-19
TLR9		transmembrane receptor	Activated	3.789	8.50E-17

**Figure 4:**
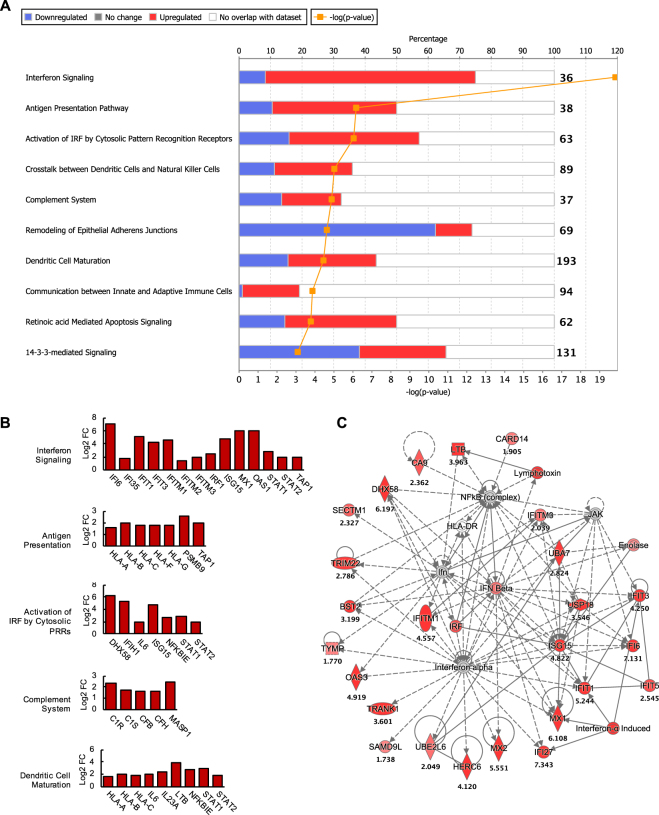
Biological pathways and functional networks modulated in ZIKV-infected hSeC at 72 hpi. (**A**) Top IPA Canonical Pathways across entire dataset at 72 hpi. Pathways order by most significant (Fisher’s exact test right-tailed). Antiviral pathways were upregulated while pathways involved in cellular organization were downregulated. Stacked bars represent percentage of total genes modulated and/or IPA predicted out of total (in bold, right) in pathway. (**B**) Significant DEGs (p < 0.05; determined by limma in Bioconductor) of antiviral, antigen presentation, and complement system pathways at 72 hpi meeting log2 FC cutoff of >|1.5|. (**C**) Top scoring IPA functional network at 72 hpi: Antimicrobial Response, Inflammatory Response, and Infectious Diseases network. Solid line between nodes represents direct interaction and dashed line represents indirect interaction. Nodes ranked by log2 FC. Node shapes represent functional classes of gene products. Vertical diamond shaped nodes represent enzymes; horizontal diamond peptidases; trapezoid transporters; triangle kinases; horizontal oval transcription regulators; vertical oval transmembrane receptors; rectangle growth factors; circle proteins. Node color indicates level of expression. Red is upregulated, and blue is downregulated. IPA functional network cutoff criteria for DEGs was log2 FC >|1.5| (p < 0.05).

The antiviral state elicited at 48 hpi was also apparent at 72 hpi. Of the top canonical pathways modulated at 72 hpi, DEGs of antiviral pathways were among those significantly upregulated (Fig. 4A). These included pathways of Interferon Signaling, Activation of IRF by Cytosolic PRRs, as well as other notable pathways, such as Antigen Presentation (Fig. 4A), which was highlighted by the upregulation of human leukocyte antigen (HLA) class I genes and genes encoding for proteasome subunit beta type-9 (*PSMB9*) and transporter associated with antigen processing 1 (*TAP1*), indicating induction of the immunoproteasome in ZIKV-infected hSeC (Fig. 4B). HLA genes were also implicated in the Dendritic Cell Maturation pathway (Fig. 4B), which included the upregulation of pro-inflammatory genes interleukin-23 subunit alpha (*IL23A*) and lymphotoxin beta (*LTB*), as well as genes overlapping with cytosolic PRR signaling such as NF-kappa-B-epsilon (*NFKBIE*), *IL6*, *STAT1*, and *STAT2*. Furthermore, complement system genes were distinctly induced in hSeC at 72 hpi (Fig. 4B). Collectively, the majority of genes and pathways induced by ZIKV at this stage of infection were linked to innate immune and inflammatory response processes. Consequently, the Antimicrobial Response, Inflammatory Response, and Infectious Diseases network was identified as the top scoring functional network at 72 hpi (Fig. 4C).

**Figure 5:**
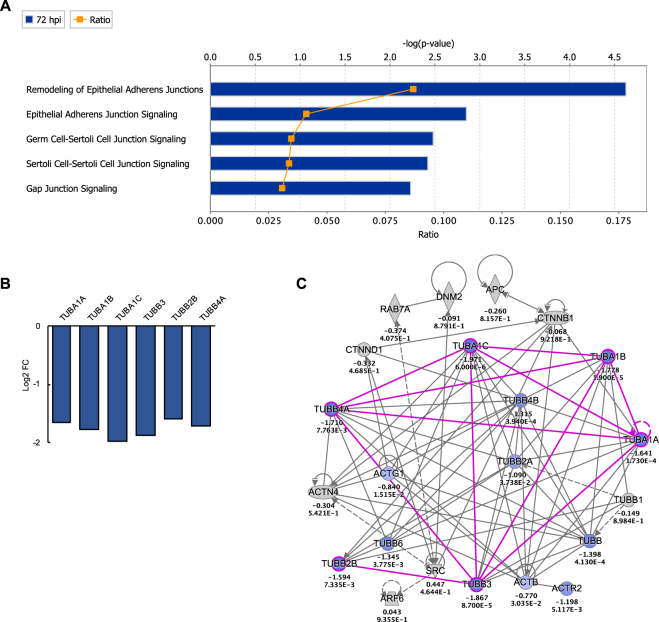
Effect of ZIKV on Cell-cell junction pathways (**A**) IPA cell-cell junction pathways modulated at 72 hpi. Ratio (orange points) represents the number of genes in a given pathway that meet the cutoff criteria (log2 FC >|1.5| and p-value of <0.05, determined by limma in Bioconductor), divided by the total number of genes that make up the pathway in the reference gene set. Blue bar represents –log(p-value). (**B**) Significant DEGs that meet the cutoff criteria among all junction pathways were those encoding for tubulin-αβ proteins. (**C**) Custom network of Remodeling of Epithelial Adherens Junctions pathway, which had the highest expression ratio among junction pathways, with 6 significant DEGs out 69 total genes in the pathway (Ratio of 0.087). Genes displayed in the network represent pathway gene coverage within the dataset. Genes such as ENDO6, TUBB, TUBB2A, TUBB4B, TUBB6, ACTB, ACTR2, and ACTG1 were significantly downregulated in the dataset (p < 0.05) but did not meet the log2 FC cutoff of >|1.5|.

In contrast, a notable pathway found to have a high proportion of downregulated genes at 72 hpi was the Remodeling of Epithelial Adherens Junctions pathway (Fig. 4A). Genes meeting the cutoff criteria (log2 FC of >|1.5|) were predominately those of tubulin (TUB) complexes, which overlapped with all cell-cell junction signaling pathways identified by IPA, including Germ Cell-Sertoli Cell Junction Signaling (Fig. 5). Moreover, similar to 12 hpi, HIST1 gene clusters, which are implicated in regulation of cellular growth and proliferation, were among the top downregulated genes at 72 hpi (Supplementary Table S3).

The top IPA predicted upstream regulators at 72 hpi, like 48 hpi, were largely associated with antiviral response, including type I (*IFNA1*, *IFNA2*, and *IFNB*) and type III (*IFNL1*) IFNs, as well as endosomal PRRs that recognize viral RNA, such as TLR3 and TLR7 (Table 3). Similar to RIG-I and MDA5, the downstream signaling of TLR3 and TLR7 involves induction of type I IFN via the activation of IRF3 or IRF7^27,28^, which were also predicted to be activated at 72 hpi (Table 3). Overall, the predicted activation of these upstream regulators is consistent with the induction of various ISGs and downstream antiviral effectors observed at this stage of infection (Figs 1E, 4B, and Supplementary Table S3). Taken together, these data suggest that peak ZIKV replication in hSeC at 72 hpi was associated with sustained induction of antiviral pathways seen at 48 hpi and repeat suppression of cellular growth and proliferation signatures observed at 12 hpi.

**Table 3:** Top 30 upstream regulators with predicted activity at 72 hpi by significance (Fisher’s exact test right-tailed).

Upstream Regulator	Expr Log Ratio	Molecule Type	Predicted Activation State	Activation z-score	p-value of overlap
IFNA2		cytokine	Activated	6.446	3.00E-48
IFNL1		cytokine	Activated	5.565	4.68E-47
Interferon alpha		group	Activated	5.87	4.54E-46
IRF7	0.998	transcription regulator	Activated	5.698	2.78E-37
PRL		cytokine	Activated	4.645	1.92E-36
MAPK1	−0.646	kinase	Inhibited	−4.861	2.10E-36
IFNG		cytokine	Activated	6.69	9.55E-35
CNOT7	−0.408	transcription regulator	Inhibited	−2.621	1.21E-34
STAT1	2.91	transcription regulator	Activated	5.339	5.97E-34
EIF2AK2	1.042	kinase	Activated	4.557	9.25E-34
NKX2-3		transcription regulator	Inhibited	−4.536	4.12E-33
IRF1	2.538	transcription regulator	Activated	4.786	2.74E-30
IRF3	0.322	transcription regulator	Activated	4.998	3.03E-26
IRF5		transcription regulator	Activated	4.349	1.73E-25
TRIM24	0.106	transcription regulator	Inhibited	−4.623	1.79E-25
IFN Beta		group	Activated	4.674	5.61E-25
Ifnar		group	Activated	4.518	7.21E-25
IL1RN		cytokine	Inhibited	−4.123	2.97E-24
Ifn		group	Activated	4.146	2.28E-23
IFNB1		cytokine	Activated	4.47	1.01E-20
IFNA1/IFNA13	0.008	cytokine	Activated	3.91	1.32E-20
MAVS	0.446	other	Activated	3.971	2.51E-20
TLR7		transmembrane receptor	Activated	4.342	7.76E-20
STAT2	1.915	transcription regulator	Activated	2.392	1.33E-19
TGM2	0.182	enzyme	Activated	4.757	1.52E-19
TLR3		transmembrane receptor	Activated	3.88	1.66E-19
IFNAR1	0.444	transmembrane receptor	Activated	2.769	1.30E-17
ACKR2		g-protein coupled receptor	Inhibited	−3.464	1.66E-16
TLR9		transmembrane receptor	Activated	4.358	5.52E-15
TNF		cytokine	Activated	5.929	4.85E-14

### Select DEGs and IPA predicted upstream regulators were validated by qRT-PCR

**Figure 6:**
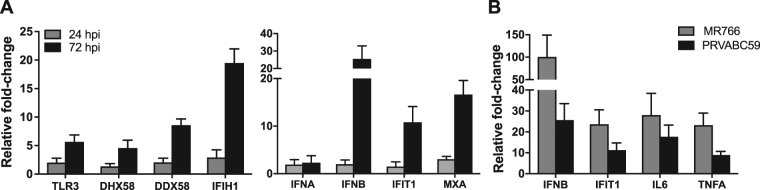
Validation of differentially expressed genes and predicted upstream regulators by qRT-PCR. hSeC were infected with ZIKV (PRVABC59 at MOI 1) and qRT-PCR was conducted on RNA extracted to validate (**A**) type I interferons and ISGs *IFIT1* and *MXA* at 24 and 72 hpi, and RNA virus-sensing PRRs *TLR3*, *DHX58* (LGP2), *DDX58* (RIG-I), and *IFIH1* (MDA5) at 24 and 72 hpi. (**B**) Validation and comparison of ISG *IFIT1* and cytokines *IL6*, and *IFNB* (IFN-β) and TNFα gene expression in hSeC at 72 hpi infected with MR766 and PRVABC59 (MOI 1). Fold-change as compared to mock was calculated by normalizing the data to GAPDH and data represents mean ± SD from at least three independent experiments.

We further validated the profile changes in select genes modulated by ZIKV infection in hSeC using quantitative RT-PCR (qRT-PCR). The changes in select antiviral genes (*IFIH1*, *IFIT1*, *IFNB*, *MXA*, *DHX58*, *DDX58*, and *TLR3*) demonstrated similar trend as seen in the RNA-seq analysis, where we observed significant upregulation only at the later time points (e.g. 72 hpi) (Fig. 6A). Similarly, the qRT-PCR data also confirmed that ZIKV did not induce significant changes in the expression of antiviral genes at 24 hpi in hSeC as compared to mock. We also confirmed whether the prototype African lineage strain of ZIKV (MR766) induces similar response as the Asian lineage strain (2015 Puerto Rico human isolate) PRVABC59 in hSeC^29^. We acknowledge that MR766 has been highly passaged in mice, whereas the clinical isolate PRVABC59 has not^29^. Further, the two strains are shown to be genetically and pathogenically distinct^29^ and thus are likely to exhibit differences in host response. However, as seen in Fig. 6B, infection of hSeC with ZIKV MR766 at the MOI of 1 also showed similar trend and induced expression of genes for IFN-β, IFIT1, and key cytokines TNF-α and IL-6 at 72 hpi.

## Discussion

Our recent study demonstrated that ZIKV productively infected primary human SC without compromising cell survival and induced multiple inflammatory mediators^14^; however, the global molecular events that occur in human SC in response to ZIKV were yet to be characterized. Here we successfully profiled the temporal transcriptomic changes in human SC following ZIKV infection to gain further insights into ZIKV infection within the human testes. Our data highlights that ZIKV suppressed signatures associated with cellular growth and proliferation during early stage of infection (12 hpi) (Figs 1 and 2), while at the later stages of infection (48 and 72 hpi), ZIKV induced signatures predominantly associated with innate antiviral defense (Figs 1, 3 and 4).

RNA viruses employ various strategies to modulate cell-cycle control and cellular proliferation in order to achieve cellular conditions conducive for virus replication^30–33^. Previous studies report downregulation of cell-cycle-related pathways in ZIKV-infected neural progenitor cells^34–38^. In addition, Garcez and colleagues reported suppression of pathways involved in chromosome replication and DNA damage and repair (DDR) in ZIKV infected neurospheres^38^. Consistent with these reports, our data identifies suppressed pathways of cellular growth and proliferation, including chromosome replication, cell-cycle control and regulation, and DDR, during early ZIKV infection of hSeC (Figs 1 and 2). However, while Tang and colleagues also noted significant dysregulation of genes associated with cell death including caspase-3 in ZIKV-human hNPC^36,39,40^, our data did not identify significant alterations in key apoptotic or cell death signatures. These results are consistent with the fact that ZIKV induces dramatic cell death in hNPC^36,41^ but not in hSeC^14^. Other *Flaviviridae*, including West Nile virus (WNV), Japanese encephalitis virus, and hepatitis C virus, are also shown to induce cell-cycle dysregulation and suppression of cellular growth and proliferation in various cell types, which is facilitated by viral hijacking of host-cell proteins that otherwise regulate host transcription and translation^31,33^. In turn, these proteins are redirected towards virus replication and assembly, depriving the host cell of normal cellular proliferative capacity^30,31,33^. Collectively, our data and previous evidence suggests that ZIKV shares a common host-cytostatic feature with related viruses in multiple cell types, including hSeC.

Upon virus infection, multiple PRRs are involved in recognizing virus components in order to elicit an innate immune response and alert neighboring cells via pro-inflammatory cytokines and IFN. Our pathway enrichment during peak ZIKV replication identified innate antiviral signaling as the top pathways modulated in hSeC (Figs 3 and 4). Pathway enrichment also identified predicted activation of RNA sensing PRRs and adapters (TLR3, TLR7, MAVS), downstream IRFs, type I and type III IFN, and pro-inflammatory cytokines (Tables 2 and 3). When compared to other flaviviruses, including ZIKV infection of other cell types, this response is not unexpected and confirms that just like other cell types, hSeC are also able to mount a robust immune response to ZIKV. Moreover, the upregulation of various ISGs at 48 and 72 hpi (Figs 1, 3 and 4), as well as the induction of the immunoproteasome at 72 hpi (Fig. 4), indicates that the cells were in an active antiviral state that included increased production of type I IFN at these stages of infection^42^. These observations were supported by the upregulation of *IFNB* at 72 hpi determined by qRT-PCR (Fig. 6), an outcome consistent with our previous study, revealing that ZIKV infection drives innate immune activation of Sertoli cells^14^. The marked induction of *IFNB* in comparison to *IFNA* may also indicate that type I IFN signaling in hSeC in response to ZIKV infection is mediated predominantly through IFN-β. Recent studies have shown that ZIKV NS5 degrades STAT2 to inhibit type I IFN signaling^43–45^. STAT2 normally functions downstream, serving as a constituent for the interferon-stimulated gene factor 3 (ISGF3) complex containing STAT1, STAT2, and IRF9, to induce expression of various ISGs, notably IRF7^46,47^. However, in our data, IRF7 was only moderately induced at 12 and 48 hpi (Supplementary Tables S1 and S2) and was not induced at 72 hpi (Supplementary Table S3). Based on these observations, it is likely that STAT2, which is shown to be induced transcriptionally in our dataset, is degraded at the protein level by ZIKV NS5 and thus may account for the biased gene expression of *IFNB* over *IFNA* in hSeC as observed in our analysis.

Studies show that ZIKV activates multiple innate immune signaling pathways, most notably through TLR3, in various cell types^34,41,48,49^. However, the literature on the ability of human SC to respond to any pathogen is very limited and no study has so far carefully characterized global changes in innate immune response in this cell type. Thus, to our knowledge, this is the first study to date providing in-depth coverage of innate antiviral responses in human SC. Available data using mouse SC show that TLRs are expressed by SC but TLR signaling is tightly regulated to control unrestrained activation of inflammatory signals^9,13,50^. Other studies found that poly(I:C), an agonist of RNA-sensing PRRs, can activate innate immune response predominantly through TLR3 in mouse SC^12,51^. Moreover, a recent report by Wu and colleagues shows that RIG-I is involved in antiviral response in mouse SC following infection with Mumps virus, providing first evidence that RIG-I may also be an important virus-sensing PRR in SC^52^. Our data supports this observation and suggests that one or more RLRs might be playing a role in detection of RNA viruses in human SC. The strong induction of IRF3-target genes, particularly *IFIT1* and *IFIT3*^15^, in our dataset may indicate that hSeC immune response to ZIKV is likely mediated by activation of IRF3 through RLR (RIG-I, MDA-5) signaling via adaptor IFN-β promoter stimulator-1 (IPS-1/MAVS) and/or TLR3 signaling via Toll/IL-1R domain-containing adaptor inducing IFN-β (TRIF)-dependent pathway^9,53^, which also indicates that TLR3 more so than TLR7 may be involved in the recognition of ZIKV in hSeC. This is also supported by the biased expression of *IFNB* over *IFNA* in our qRT-PCR analysis, as for other flaviviruses, such as WNV, IFN-α-dependent control of WNV infection in certain cell types requires IRF7^54^, which is activated distinctly by TLR7/8/9^46^, whereas IFN-β can be induced independently of IRF7 through IRF3^46,54^. However, additional functional studies are warranted to carefully delineate the relative contribution of viral RNA-sensing PRRs in the antiviral defense response of human SC.

Recent studies have identified TAM (Tyro3, Axl, Mer) receptors, especially Axl, as virus entry receptors for ZIKV in a cell-type-specific manner^48,49,55–57^. While Axl has been shown to facilitate ZIKV entry in many cell types, including human skin cells^48^, human endothelial cells^56,57^, and human glial cells^49^, it was found to be dispensable in hNPC and cerebral organoids^55^. Recent reports also provide evidence of the immunomodulatory role of Axl during ZIKV infection. Chen and colleagues recently showed that Axl was involved in antagonizing IFN signaling and not ZIKV entry in human astrocytes^58^. Furthermore, Axl is shown to be dispensable for ZIKV infection in IFNAR-deficient mice^59,60^. Taken together, these findings indicate that the role of Axl in ZIKV infection may be cell-type-specific and is potentially linked to fully functional IFNAR, and thus remains controversial. Although data in human SC is lacking, it is reported that mouse SC stimulated with TLR3 agonist poly(I:C) exhibit increased levels of Axl expression^51^. Our data however, did not identify induction of any TAM receptor genes in hSeC following ZIKV infection but predicted activation of TGF-β signaling at 12 hpi (Table 1). Interestingly, SC are one of few cell types known to constitutively express high levels of TAM receptors that, along with TGF-β and activin A, play an important role in maintaining the immunosuppressive milieu of seminiferous tubules^9^. Therefore, it is likely that although TAM receptors are not further induced by ZIKV in these cells, they still may act as ZIKV-entry receptors and/or immunomodulators by dampening PRR signaling^61^. The TGF-β family of proteins are also known to regulate blood-testis barrier integrity and germ cell differentiation under physiological conditions^9^. Thus, collectively, the role of TAM receptors and TGF-β family of proteins in modulating the testicular immune environment and spermatogenesis during ZIKV infection warrants closer examination.

Interestingly, pathway enrichment of late ZIKV infection (72 hpi) in hSeC identified downregulation of pathways associated with adherens junctions remodeling (Fig. 5), which are also implicated in germ cell-Sertoli cell junction signaling^62^. A recent report by McGrath and colleagues identified similar suppression of tubulin, specifically tubulin-β class III (*TUBB3*), in primary hNPC post-ZIKV infection^37^. Furthermore, the group identified suppression of histone cluster 1 family genes in ZIKV-infected hNPC^37^, which we also found in infected hSeC at both 12 hpi and 72 hpi (Fig. 2; Supplementary Tables S1 and S3). Tubulin-α/β are the dimer subunits of microtubules, the major components of the cytoskeleton that serve various functions, including cell structural support, intracellular transport, and chromosome segregation^63^. Microtubules are also actively manipulated by viruses, including ZIKV, during infection to facilitate virus entry, trafficking, and replication^64,65^. Histone cluster 1 family proteins are implicated in various processes of cell-cycle progression, including transcriptional regulation, DNA replication and repair, and chromosomal architecture^66,67^. Therefore, the suppression of TUBA/B and HIST1 genes by ZIKV infection in hSeC provides further evidence that ZIKV replication promotes cytostatic effects in multiple cell types upon infection.

Other signaling pathways of interest modulated at 12 hpi and 72 hpi include high mobility group box 1 (HGMB1) Signaling and Complement System pathways, respectively (Figs 2A and 4A). HMGB1 signaling has been implicated in flavivirus infection, particularly DENV^68,69^. HMGB1 protein, normally located in the nucleosome, is released actively by macrophages/monocytes during inflammation or passively by necrotic cells and modulates a broad range of immunological responses upon exogenous recognition by TLR2, TLR4, and/or the receptor for advanced glycation end products (RAGE; *AGER*)^68–70^. Interestingly, HMGB1 signaling is also described in SC with RAGE as the primary receptor for HMGB1^70^ and is associated with chronic inflammation and autoimmunity in the testes^70^. However, although induction of HMGB1 signaling is consistent with flavivirus infections, its functional role in context of ZIKV-infected hSeC remains to be determined. In contrast to HMGB1 signaling, to our knowledge, this study is first to demonstrate induction of complement system gene expression in SC. Complement system proteins are an integral part of the innate immune system that play a crucial role in eliminating extracellular pathogens and necrotic/apoptotic cells^71^. The majority of these proteins are traditionally known to be produced by the liver; however, local tissue production is reported to be the main source of complement in the immune-privileged sites of the brain and eye^71^ and may be induced in these sites by pro-inflammatory stimuli^72^. Accordingly, it is likely that SC are a source of complement under normal physiological conditions, and although the upregulation of complement genes is probably a byproduct of increased cytokine levels due to ZIKV infection, the mechanisms underlying their induction in SC require further investigation.

In summary, our results fill a major gap in our understanding of host-ZIKV interaction in an important cell-type within the immune privileged compartment of the testis. Clearly, ZIKV takes advantage of the immunosuppressive nature of SC and demonstrates modulation of host antiviral response and cellular proliferation pathways to establish infection. Currently, little is known of the mechanisms of ZIKV persistence in the human testes. Why despite the strong antiviral response in human SC demonstrated here does the virus then persist in these cells^14^ and in the testes, which is evident by the prolonged detection of the virus in semen and spermatozoa^2,3,5^? A possible explanation might be that alterations in other negative regulators of innate immune pathways such as TGF-β and TAM receptor signaling in hSeC may allow infected cells to survive longer as compared to other cellular targets including NPC^39,41^, thus contributing to persistence. Accordingly, future studies focusing on systematic attenuation of these signaling pathways in hSeC may better elucidate their role in ZIKV persistence. Moreover, an increase in the TNF and IL-6 families of cytokines may have implications on other important testicular cell functions during ZIKV infection, including steroidogenesis and leukocyte transmigration, and therefore further investigation in this area is also warranted. Importantly, this study is first to report enrichment of multiple innate immune pathways in human SC, indicating that these cells are capable of activating strong antiviral defense to ZIKV and possibly other testes-tropic viruses including Ebola virus. Thus, these data will serve as a framework for future investigations of specific cellular and molecular mechanisms underlying virus infection and persistence, as well as crosstalk with other resident cell types of the testes.

## Materials and Methods

### Cells and virus

Low-passage primary human Sertoli cells (hSeC) purchased from Lonza (MM-HSE-2305) were propagated in DMEM/F-12 media (ratio of 1:1) with HEPES, L-Gln, 100 units/mL Pen-Strep, and 5% untreated FBS as described previously^14^. All experiments were conducted using passage 6–9 at 80–90% confluency. ZIKV strain PRVABC59 (Human/2015/Puerto Rico) acquired from American Type Culture Collection (ATCC) was propagated once in Vero E6 cells (ATCC). Stock virus titer was measured by plaque assay using Vero cells to calculate MOI for infection. Cells were infected at MOI 1 for 1 hr at 37 °C followed by 2 washes and replaced with fresh media^14^. Cytopathic effect was assessed using the CellTiter 96 AQueous One Solution cell proliferation assay kit according to manufacturer specifications, and percent cell viability was calculated by comparison to mock-infected cells at corresponding time point. For quantification, ZIKV titers in cell culture supernatants were analyzed by a standard plaque assay using Vero cells as described previously^14^; data expressed as ZIKV PFU/mL supernatant.

### RNA sample prep and library construction

Total RNA was purified from ZIKV (PRVABC59)-infected hSeC at 12, 24, 48, and 72 hours after infection as well as from mock-infected hSeC at 24 hours (control), in triplicates for each time point, using a RNeasy kit (QIAGEN). RNA was assessed using a Bioanalyzer (Agilent, USA) for RNA integrity number (RIN) of ≥8 and sent to Seattle Genomics (University of Washington) for cDNA library construction and next-generation sequencing (Total RNA-seq) using an Illumina Nextseq. 500 instrument.

### RNA-seq data processing and analysis

Raw RNA-seq data (Fastq files) were demultiplexed, checked for quality (FastQC version 0.11.3), rRNA was then digitally removed using Bowtie2 (version 2.2.5). Sufficient host reads (~30 million) were then mapped to the human genome (NCBI 37.1) using STAR (2.4.2) and then converted into gene counts with ht-seq (0.6.0). Both the genome sequence (fasta) and gene transfer files (gtf) for human were obtained from the igenomes website (https://support.illumina.com/sequencing/sequencing_software/igenome.html).

Gene counts were then loaded in the R statistical programming language (version 3.2.0) and filtered by a mean of ten or greater across all samples. Viral alignment was performed using Bowtie2 (version 2.2.5) against ZIKV sequence (Genbank KU501215.1). Default alignment parameters were used. Exploratory analysis and statistics were also run using R and Bioconductor. The gene count matrix was normalized using voom through the limma Bioconductor package (3.26.9). Statistical analysis (including differential expression) was performed using the limma Bioconductor package. For heatmap generation, the union of differentially expressed genes were then loaded into a custom R function using gplots and WGCNA Bioconductor packages. In order for a gene to be considered part of the union it needed a log2 fold-change of >|1.5| and an adjusted p-value of <0.05 in each of the conditions. Clustering was performed on the fold change ratios of co-regulated gene modules were identified using the WGCNA package.

### Functional analysis

Ingenuity Pathway Analysis (IPA) was used to determine enrichment of biological functions and networks. IPA produced functional networks, pathways, and predicted upstream transcriptional regulators. IPA cutoff criteria for input list of differentially expressed genes was set to log2 fold-change >|1.5| and adjusted p-value of <0.05. Stacked bar charts represent most significant Canonical Pathways determined by the cutoff criteria across the entire dataset. The significance values for canonical pathways were calculated by Fisher’s exact test right-tailed. The significance indicates the probability of association of molecules from the dataset with the canonical pathway by random chance alone.

### Validation by qRT-PCR

Total RNA extracted from hSeC at different time points after ZIKV infection was used to measure changes in the transcripts of multiple innate immune genes by qRT-PCR using specific primers (Supplementary Table S4). The housekeeping gene GAPDH was used to normalize fold-change of each gene as compared to mock-infected control and was calculated as described previously^14^.

## Electronic supplementary material

Dataset 1
