# Supplementary material for: Immunoprofiles of human Sertoli cells infected with Zika virus reveals unique insights into host-pathogen crosstalk
Source: Sci Rep. 2018 Jun 7;8:8702. doi: 10.1038/s41598-018-27027-7 (PMC5992156; doi:10.1038/s41598-018-27027-7)
Supplement: Supplementary file 1 — Dataset 1 [file 41598_2018_27027_MOESM1_ESM.docx]

**Immunoprofiles of human Sertoli cells infected with Zika virus reveals unique insights into host-pathogen crosstalk**

Daniel P. Strange^1^, Richard Green^2^, David N. Siemann^1^, Michael Gale Jr.^2^* and Saguna Verma^1^*

1 Department of Tropical Medicine, Medical Microbiology and Pharmacology, John A. Burns School of Medicine, University of Hawaii at Manoa, Honolulu, Hawaii

2 Department of Immunology, Center for Innate Immunity and Immune Disease, University of Washington School of Medicine, Seattle, Washington

*Corresponding authors: mgale@uw.edu; saguna@hawaii.edu

**Supplementary Table S1.** Differentially expressed genes (p<0.05) of ZIKV-infected hSeC at 12 hpi. Ranked by Log2 FC.

| **Gene Symbol** | **Gene Name** | **Log2 FC** | **p-value** |
| --- | --- | --- | --- |
| TNS4 | tensin 4 | 5.438 | 2.10E-04 |
| NPPB | natriuretic peptide B | 4.765 | 3.82E-02 |
| L3HYPDH | trans-L-3-hydroxyproline dehydratase | 4.06 | 1.30E-05 |
| IL11 | interleukin 11 | 3.904 | 7.95E-04 |
| LMCD1 | LIM and cysteine rich domains 1 | 3.888 | 4.16E-03 |
| HES4 | hes family bHLH transcription factor 4 | 3.526 | 1.45E-02 |
| MOCS3 | molybdenum cofactor synthesis 3 | 3.377 | 2.54E-02 |
| IL6 | interleukin 6 | 2.707 | 2.67E-03 |
| PTGIS | prostaglandin I2 synthase | 2.665 | 3.11E-02 |
| LIMCH1 | LIM and calponin homology domains 1 | 2.661 | 2.10E-02 |
| WNT2B | Wnt family member 2B | 2.604 | 2.69E-02 |
| SCMH1 | sex comb on midleg homolog 1 (Drosophila) | 2.444 | 2.22E-03 |
| F3 | coagulation factor III, tissue factor | 2.268 | 1.85E-03 |
| ZNF469 | zinc finger protein 469 | 2.255 | 1.62E-03 |
| FTCD | formimidoyltransferase cyclodeaminase | 2.237 | 1.51E-03 |
| PTGS2 | prostaglandin-endoperoxide synthase 2 | 2.196 | 1.08E-03 |
| LIF | leukemia inhibitory factor | 2.194 | 4.16E-03 |
| SPHK1 | sphingosine kinase 1 | 2.152 | 7.95E-04 |
| TM4SF1 | transmembrane 4 L six family member 1 | 2.139 | 1.24E-03 |
| ICAM1 | intercellular adhesion molecule 1 | 2.136 | 8.56E-03 |
| CBARP | CACN beta subunit associated regulatory protein | 2.078 | 1.31E-02 |
| FAAP100 | Fanconi anemia core complex associated protein 100 | 2.051 | 3.85E-04 |
| ACTA2 | actin, alpha 2, smooth muscle, aorta | 2.032 | 4.67E-03 |
| ZFC3H1 | zinc finger C3H1-type containing | 2.029 | 3.90E-05 |
| PLOD2 | procollagen-lysine,2-oxoglutarate 5-dioxygenase 2 | 1.99 | 3.69E-03 |
| INHBA | inhibin beta A subunit | 1.973 | 5.96E-04 |
| IL33 | interleukin 33 | 1.952 | 1.09E-03 |
| MYO7B | myosin VIIB | 1.917 | 2.66E-03 |
| CRISPLD2 | cysteine rich secretory protein LCCL domain containing 2 | 1.884 | 3.78E-02 |
| SH2D4A | SH2 domain containing 4A | 1.883 | 1.08E-03 |
| SMOX | spermine oxidase | 1.834 | 1.45E-02 |
| SPAG4 | sperm associated antigen 4 | 1.828 | 6.95E-03 |
| FGF2 | fibroblast growth factor 2 | 1.813 | 4.73E-03 |
| ST7L | suppression of tumorigenicity 7 like | 1.805 | 2.59E-03 |
| CCL2 | C-C motif chemokine ligand 2 | 1.769 | 8.58E-03 |
| SLC7A5 | solute carrier family 7 member 5 | 1.761 | 6.12E-03 |
| DPYSL4 | dihydropyrimidinase like 4 | 1.757 | 3.41E-02 |
| VGLL3 | vestigial like family member 3 | 1.745 | 4.28E-02 |
| HSPB7 | heat shock protein family B (small) member 7 | 1.725 | 1.78E-02 |
| GPRC5A | G protein-coupled receptor class C group 5 member A | 1.686 | 3.28E-02 |
| HEG1 | heart development protein with EGF like domains 1 | 1.674 | 8.04E-03 |
| PCSK7 | proprotein convertase subtilisin/kexin type 7 | 1.663 | 4.03E-03 |
| NTN3 | netrin 3 | 1.637 | 6.13E-03 |
| TNFAIP3 | TNF alpha induced protein 3 | 1.625 | 9.69E-04 |
| EGR1 | early growth response 1 | 1.622 | 7.22E-03 |
| EPPK1 | epiplakin 1 | 1.619 | 2.13E-02 |
| NDE1 | nudE neurodevelopment protein 1 | 1.612 | 4.22E-03 |
| MEDAG | mesenteric estrogen dependent adipogenesis | 1.579 | 2.78E-02 |
| KCNK6 | potassium two pore domain channel subfamily K member 6 | 1.57 | 3.77E-02 |
| COL4A1 | collagen type IV alpha 1 chain | 1.561 | 3.00E-02 |
| EHD1 | EH domain containing 1 | 1.559 | 4.78E-03 |
| CSNK1D | casein kinase 1 delta | 1.543 | 9.69E-04 |
| SIRPA | signal regulatory protein alpha | 1.542 | 2.29E-02 |
| MAP3K4 | mitogen-activated protein kinase kinase kinase 4 | 1.52 | 2.67E-03 |
| VEGFA | vascular endothelial growth factor A | 1.519 | 3.48E-03 |
| BNIP3L | BCL2 interacting protein 3 like | 1.515 | 4.80E-02 |
| KDM6B | lysine demethylase 6B | 1.513 | 4.28E-02 |
| CD2AP | CD2 associated protein | 1.488 | 3.41E-02 |
| ANKZF1 | ankyrin repeat and zinc finger domain containing 1 | 1.446 | 2.30E-02 |
| BMP2 | bone morphogenetic protein 2 | 1.433 | 7.95E-04 |
| ANKLE2 | ankyrin repeat and LEM domain containing 2 | 1.412 | 9.68E-03 |
| FAM167A | family with sequence similarity 167 member A | 1.407 | 4.60E-02 |
| ZNF503 | zinc finger protein 503 | 1.403 | 9.86E-04 |
| CNN1 | calponin 1 | 1.396 | 3.94E-02 |
| SLFNL1 | schlafen like 1 | 1.376 | 1.35E-02 |
| AKAP12 | A-kinase anchoring protein 12 | 1.363 | 3.00E-02 |
| CSRP1 | cysteine and glycine rich protein 1 | 1.348 | 2.60E-02 |
| KCNC4 | potassium voltage-gated channel subfamily C member 4 | 1.317 | 2.13E-02 |
| ITPRIP | inositol 1,4,5-trisphosphate receptor interacting protein | 1.312 | 1.22E-02 |
| CALD1 | caldesmon 1 | 1.303 | 1.28E-02 |
| FEM1B | fem-1 homolog B | 1.296 | 2.69E-02 |
| FGFR1 | fibroblast growth factor receptor 1 | 1.294 | 2.99E-02 |
| MYO10 | myosin X | 1.276 | 1.08E-03 |
| PQLC1 | PQ loop repeat containing 1 | 1.264 | 4.54E-02 |
| FOSB | FosB proto-oncogene, AP-1 transcription factor subunit | 1.23 | 4.60E-02 |
| GYS1 | glycogen synthase 1 | 1.212 | 3.67E-02 |
| HIP1R | huntingtin interacting protein 1 related | 1.192 | 4.71E-02 |
| OPA3 | OPA3, outer mitochondrial membrane lipid metabolism regulator | 1.134 | 3.86E-03 |
| TUBB2A | tubulin beta 2A class IIa | 1.128 | 4.47E-02 |
| TAS1R3 | taste 1 receptor member 3 | 0.907 | 3.11E-02 |
| SZT2 | seizure threshold 2 homolog (mouse) | -0.656 | 4.80E-02 |
| AEBP1 | AE binding protein 1 | -0.781 | 3.16E-02 |
| NRP1 | neuropilin 1 | -0.803 | 2.60E-02 |
| MMEL1 | membrane metalloendopeptidase like 1 | -0.813 | 3.65E-02 |
| TNS2 | tensin 2 | -0.854 | 3.22E-02 |
| LMNB2 | lamin B2 | -0.866 | 3.22E-02 |
| KHNYN | KH and NYN domain containing | -0.951 | 4.91E-02 |
| FUT8 | fucosyltransferase 8 | -0.96 | 3.90E-02 |
| MEA1 | male-enhanced antigen 1 | -0.966 | 2.91E-02 |
| KANK2 | KN motif and ankyrin repeat domains 2 | -0.971 | 3.22E-02 |
| SLIT2 | slit guidance ligand 2 | -1.008 | 4.97E-02 |
| HNRNPA2B1 | heterogeneous nuclear ribonucleoprotein A2/B1 | -1.012 | 4.28E-02 |
| PTMA | prothymosin, alpha | -1.021 | 1.63E-02 |
| DNMT1 | DNA methyltransferase 1 | -1.033 | 3.86E-03 |
| FAN1 | FANCD2 and FANCI associated nuclease 1 | -1.05 | 4.80E-02 |
| UACA | uveal autoantigen with coiled-coil domains and ankyrin repeats | -1.066 | 3.81E-02 |
| CD27 | CD27 molecule | -1.094 | 3.08E-02 |
| CHRNE | cholinergic receptor nicotinic epsilon subunit | -1.099 | 4.80E-02 |
| WDR6 | WD repeat domain 6 | -1.115 | 3.67E-02 |
| FAM151A | family with sequence similarity 151 member A | -1.136 | 3.94E-02 |
| MTHFD1 | methylenetetrahydrofolate dehydrogenase, cyclohydrolase and formyltetrahydrofolate synthetase 1 | -1.137 | 3.79E-02 |
| SLC25A35 | solute carrier family 25 member 35 | -1.141 | 1.65E-02 |
| NCAPD2 | non-SMC condensin I complex subunit D2 | -1.156 | 4.31E-02 |
| SSBP4 | single stranded DNA binding protein 4 | -1.158 | 2.88E-02 |
| CEMP1 | cementum protein 1 | -1.19 | 3.34E-03 |
| POLG | DNA polymerase gamma, catalytic subunit | -1.212 | 2.21E-03 |
| COL13A1 | collagen type XIII alpha 1 chain | -1.237 | 3.11E-02 |
| LYPD1 | LY6/PLAUR domain containing 1 | -1.242 | 2.13E-02 |
| PARP1 | poly(ADP-ribose) polymerase 1 | -1.257 | 2.10E-02 |
| HMGN2 | high mobility group nucleosomal binding domain 2 | -1.265 | 2.68E-02 |
| ANXA9 | annexin A9 | -1.273 | 4.71E-02 |
| MCM7 | minichromosome maintenance complex component 7 | -1.281 | 3.11E-02 |
| NSD2 | nuclear receptor binding SET domain protein 2 | -1.301 | 3.50E-03 |
| ESRRA | estrogen related receptor alpha | -1.317 | 4.85E-02 |
| DEK | DEK proto-oncogene | -1.324 | 7.95E-04 |
| ADCY3 | adenylate cyclase 3 | -1.327 | 4.67E-03 |
| ZNF276 | zinc finger protein 276 | -1.328 | 3.64E-03 |
| HMGA1 | high mobility group AT-hook 1 | -1.336 | 7.95E-04 |
| MCM2 | minichromosome maintenance complex component 2 | -1.346 | 3.58E-02 |
| SMC4 | structural maintenance of chromosomes 4 | -1.348 | 1.88E-02 |
| UHRF1 | ubiquitin like with PHD and ring finger domains 1 | -1.349 | 1.18E-02 |
| TMPO | thymopoietin | -1.351 | 2.92E-02 |
| PAQR5 | progestin and adipoQ receptor family member 5 | -1.357 | 3.11E-02 |
| CHAF1A | chromatin assembly factor 1 subunit A | -1.373 | 1.09E-02 |
| E2F1 | E2F transcription factor 1 | -1.381 | 4.75E-02 |
| HIRIP3 | HIRA interacting protein 3 | -1.417 | 2.13E-02 |
| RFXANK | regulatory factor X associated ankyrin containing protein | -1.418 | 5.75E-03 |
| SDC1 | syndecan 1 | -1.456 | 1.86E-02 |
| ATG7 | autophagy related 7 | -1.459 | 3.78E-02 |
| STEAP3 | STEAP3 metalloreductase | -1.468 | 1.33E-02 |
| INCENP | inner centromere protein | -1.508 | 7.95E-04 |
| TIAM2 | T-cell lymphoma invasion and metastasis 2 | -1.553 | 3.41E-02 |
| CDC42EP4 | CDC42 effector protein 4 | -1.556 | 3.41E-02 |
| SOX12 | SRY-box 12 | -1.594 | 1.78E-03 |
| TRERF1 | transcriptional regulating factor 1 | -1.644 | 4.74E-02 |
| CCNA2 | cyclin A2 | -1.647 | 2.07E-02 |
| GPR39 | G protein-coupled receptor 39 | -1.684 | 1.51E-03 |
| HIST4H4 | histone cluster 4 H4 | -1.685 | 2.69E-02 |
| PKMYT1 | protein kinase, membrane associated tyrosine/threonine 1 | -1.692 | 9.69E-04 |
| GPSM2 | G-protein signaling modulator 2 | -1.701 | 1.84E-02 |
| HIST1H2BK | histone cluster 1 H2B family member k | -1.704 | 7.95E-04 |
| THRA | thyroid hormone receptor, alpha | -1.715 | 4.60E-05 |
| H2AFX | H2A histone family member X | -1.717 | 2.06E-04 |
| MMP19 | matrix metallopeptidase 19 | -1.72 | 6.03E-03 |
| CLCC1 | chloride channel CLIC like 1 | -1.735 | 2.93E-02 |
| LCTL | lactase like | -1.743 | 3.06E-03 |
| PRC1 | protein regulator of cytokinesis 1 | -1.767 | 3.25E-02 |
| MCM4 | minichromosome maintenance complex component 4 | -1.769 | 2.02E-02 |
| ARL4C | ADP ribosylation factor like GTPase 4C | -1.811 | 2.08E-02 |
| HIST2H2AC | histone cluster 2 H2A family member c | -1.813 | 6.03E-03 |
| HIST1H2BC | histone cluster 1 H2B family member c | -1.82 | 2.40E-04 |
| MCM5 | minichromosome maintenance complex component 5 | -1.833 | 5.92E-03 |
| KPNA2 | karyopherin subunit alpha 2 | -1.866 | 3.14E-03 |
| FLG | filaggrin | -1.87 | 2.12E-02 |
| MLX | MLX, MAX dimerization protein | -1.876 | 9.09E-03 |
| NCAPD3 | non-SMC condensin II complex subunit D3 | -1.891 | 3.42E-02 |
| KIFC1 | kinesin family member C1 | -1.913 | 2.10E-02 |
| TRHDE | thyrotropin releasing hormone degrading enzyme | -1.917 | 9.69E-04 |
| HIST2H2BE | histone cluster 2 H2B family member e | -1.927 | 1.62E-03 |
| NASP | nuclear autoantigenic sperm protein | -1.975 | 3.00E-02 |
| SLC29A2 | solute carrier family 29 member 2 | -1.975 | 3.90E-02 |
| TCF19 | transcription factor 19 | -1.988 | 6.32E-03 |
| FOXM1 | forkhead box M1 | -1.99 | 6.08E-03 |
| HMGB2 | high mobility group box 2 | -2.013 | 2.24E-03 |
| LSMEM2 | leucine rich single-pass membrane protein 2 | -2.026 | 1.48E-02 |
| HIST1H2BJ | histone cluster 1 H2B family member j | -2.027 | 2.10E-02 |
| PREX1 | phosphatidylinositol-3,4,5-trisphosphate dependent Rac exchange factor 1 | -2.065 | 3.11E-02 |
| HIST1H2AE | histone cluster 1 H2A family member e | -2.071 | 6.47E-03 |
| TACC3 | transforming acidic coiled-coil containing protein 3 | -2.092 | 7.29E-04 |
| LIMD2 | LIM domain containing 2 | -2.108 | 1.70E-03 |
| UBE2C | ubiquitin conjugating enzyme E2 C | -2.108 | 5.97E-03 |
| CDC20 | cell division cycle 20 | -2.109 | 1.21E-02 |
| HIST1H2BG | histone cluster 1 H2B family member g | -2.116 | 5.30E-03 |
| IPPK | inositol-pentakisphosphate 2-kinase | -2.202 | 4.55E-03 |
| MYBL2 | MYB proto-oncogene like 2 | -2.207 | 2.67E-03 |
| TSHZ1 | teashirt zinc finger homeobox 1 | -2.232 | 4.28E-02 |
| HIST1H1C | histone cluster 1 H1 family member c | -2.239 | 6.00E-06 |
| NES | nestin | -2.271 | 3.69E-03 |
| HIST1H1E | histone cluster 1 H1 family member e | -2.29 | 1.30E-05 |
| VSIG2 | V-set and immunoglobulin domain containing 2 | -2.293 | 4.60E-02 |
| PAQR4 | progestin and adipoQ receptor family member 4 | -2.329 | 8.04E-03 |
| KIF11 | kinesin family member 11 | -2.334 | 1.25E-02 |
| HIST1H2AH | histone cluster 1 H2A family member h | -2.367 | 1.31E-02 |
| HIST1H4D | histone cluster 1 H4 family member d | -2.378 | 9.09E-03 |
| PLK1 | polo like kinase 1 | -2.395 | 1.78E-02 |
| KIF18B | kinesin family member 18B | -2.396 | 1.22E-02 |
| LIG1 | DNA ligase 1 | -2.399 | 2.20E-03 |
| MRPL57 | mitochondrial ribosomal protein L57 | -2.419 | 7.72E-04 |
| MMP1 | matrix metallopeptidase 1 | -2.457 | 4.00E-05 |
| TPX2 | TPX2, microtubule nucleation factor | -2.461 | 7.49E-04 |
| CENPF | centromere protein F | -2.499 | 3.50E-03 |
| HIST1H2BD | histone cluster 1 H2B family member d | -2.575 | 3.90E-05 |
| HIST1H2AK | histone cluster 1 H2A family member k | -2.599 | 3.06E-02 |
| GTSE1 | G2 and S-phase expressed 1 | -2.608 | 7.12E-03 |
| HIST2H2BF | histone cluster 2 H2B family member f | -2.613 | 1.08E-03 |
| TYMSOS | TYMS opposite strand | -2.618 | 4.00E-05 |
| SCYL3 | SCY1 like pseudokinase 3 | -2.651 | 2.37E-02 |
| HIST1H3I | histone cluster 1 H3 family member i | -2.658 | 7.95E-04 |
| HIST1H2BE | histone cluster 1 H2B family member e | -2.666 | 1.97E-04 |
| HIST1H2BL | histone cluster 1 H2B family member l | -2.677 | 2.82E-03 |
| MKI67 | marker of proliferation Ki-67 | -2.706 | 3.90E-05 |
| PER2 | period circadian clock 2 | -2.722 | 4.58E-02 |
| CLSPN | claspin | -2.766 | 3.52E-02 |
| HIST1H3H | histone cluster 1 H3 family member h | -2.795 | 2.10E-04 |
| HIST1H2AM | histone cluster 1 H2A family member m | -2.797 | 8.98E-03 |
| HIST1H3D | histone cluster 1 H3 family member d | -2.826 | 1.97E-04 |
| HIST2H3D | histone cluster 2 H3 family member d | -2.83 | 1.51E-03 |
| HIST1H2AL | histone cluster 1 H2A family member l | -2.88 | 2.20E-03 |
| HIST1H2BN | histone cluster 1 H2B family member n | -2.919 | 9.95E-04 |
| HIST1H3B | histone cluster 1 H3 family member b | -2.991 | 3.90E-05 |
| CKAP2L | cytoskeleton associated protein 2 like | -3.006 | 2.54E-02 |
| CIT | citron rho-interacting serine/threonine kinase | -3.065 | 3.64E-03 |
| HIST1H1D | histone cluster 1 H1 family member d | -3.07 | 1.97E-04 |
| KIF4A | kinesin family member 4A | -3.082 | 3.82E-02 |
| HIST1H2AI | histone cluster 1 H2A family member i | -3.173 | 2.06E-04 |
| HIST1H2BM | histone cluster 1 H2B family member m | -3.201 | 3.94E-03 |
| ANLN | anillin actin binding protein | -3.209 | 1.08E-03 |
| LCORL | ligand dependent nuclear receptor corepressor like | -3.246 | 5.44E-03 |
| TOP2A | topoisomerase (DNA) II alpha | -3.314 | 2.20E-03 |
| HIST1H3A | histone cluster 1 H3 family member a | -3.34 | 1.85E-03 |
| HIST3H2BB | histone cluster 3 H2B family member b | -3.341 | 3.41E-02 |
| ENOSF1 | enolase superfamily member 1 | -3.351 | 2.60E-03 |
| HIST1H2BF | histone cluster 1 H2B family member f | -3.351 | 8.52E-03 |
| HIST1H2BO | histone cluster 1 H2B family member o | -3.428 | 8.01E-04 |
| HIST2H3A | histone cluster 2 H3 family member a | -3.514 | 7.29E-04 |
| ARHGAP11A | Rho GTPase activating protein 11A | -3.525 | 2.27E-02 |
| HJURP | Holliday junction recognition protein | -3.581 | 1.23E-02 |
| HIST1H1B | histone cluster 1 H1 family member b | -3.639 | 0.00E+00 |
| C10orf55 | chromosome 10 open reading frame 55 | -3.649 | 3.90E-05 |
| HIST1H2AJ | histone cluster 1 H2A family member j | -3.668 | 3.77E-04 |
| BUB1B | BUB1 mitotic checkpoint serine/threonine kinase B | -3.677 | 1.29E-02 |
| HIST1H2AG | histone cluster 1 H2A family member g | -3.718 | 1.92E-04 |
| SPC24 | SPC24, NDC80 kinetochore complex component | -3.745 | 1.57E-02 |
| KIAA0895 | KIAA0895 | -3.756 | 9.36E-04 |
| TRIM59 | tripartite motif containing 59 | -3.822 | 1.93E-02 |
| HIST1H2BI | histone cluster 1 H2B family member i | -3.892 | 1.09E-03 |
| COL22A1 | collagen type XXII alpha 1 chain | -3.908 | 5.30E-03 |
| ANKRD55 | ankyrin repeat domain 55 | -3.934 | 1.16E-02 |
| PLAU | plasminogen activator, urokinase | -3.965 | 8.32E-04 |
| HIST2H3C | histone cluster 2 H3 family member c | -4.046 | 1.67E-03 |
| APOL3 | apolipoprotein L3 | -4.053 | 1.53E-02 |
| STMN1 | stathmin 1 | -4.067 | 9.95E-03 |
| HIST1H3J | histone cluster 1 H3 family member j | -4.219 | 2.93E-03 |
| IQGAP3 | IQ motif containing GTPase activating protein 3 | -4.268 | 8.11E-04 |
| HIST1H3G | histone cluster 1 H3 family member g | -4.291 | 1.65E-04 |
| ZWINT | ZW10 interacting kinetochore protein | -4.383 | 1.72E-03 |
| HIST1H4L | histone cluster 1 H4 family member l | -4.436 | 9.97E-04 |
| PCNA | proliferating cell nuclear antigen | -4.618 | 1.70E-04 |
| HIST1H3C | histone cluster 1 H3 family member c | -4.674 | 1.70E-03 |
| NUSAP1 | nucleolar and spindle associated protein 1 | -5.645 | 6.10E-05 |
| HIST1H1A | histone cluster 1 H1 family member a | -5.83 | 2.61E-04 |

**Supplementary Table S2.** Differentially expressed genes (p<0.05) of ZIKV-infected hSeC at 48 hpi. Ranked by Log2 FC.

| **Gene Symbol** | **Gene Name** | **Log2 FC** | **p-value** |
| --- | --- | --- | --- |
| IFI27 | interferon alpha inducible protein 27 | 6.634 | 3.44E-04 |
| OAS1 | 2'-5'-oligoadenylate synthetase 1 | 6.424 | 7.85E-04 |
| MX1 | MX dynamin like GTPase 1 | 6.318 | 4.30E-05 |
| EPSTI1 | epithelial stromal interaction 1 | 6.123 | 3.87E-04 |
| DHX58 | DExH-box helicase 58 | 6.099 | 1.21E-04 |
| IFI6 | interferon alpha inducible protein 6 | 5.876 | 2.00E-05 |
| MX2 | MX dynamin like GTPase 2 | 5.824 | 6.95E-03 |
| IFIT1 | interferon induced protein with tetratricopeptide repeats 1 | 5.693 | 4.39E-02 |
| OASL | 2'-5'-oligoadenylate synthetase like | 5.658 | 5.16E-03 |
| BATF2 | basic leucine zipper ATF-like transcription factor 2 | 5.588 | 2.39E-03 |
| CMPK2 | cytidine/uridine monophosphate kinase 2 | 5.508 | 4.07E-04 |
| IFI44L | interferon induced protein 44 like | 5.464 | 3.25E-02 |
| FNDC11 | fibronectin type III domain containing 11 | 5.432 | 2.71E-03 |
| ATF3 | activating transcription factor 3 | 5.371 | 1.07E-03 |
| IFIH1 | interferon induced with helicase C domain 1 | 5.252 | 1.95E-03 |
| OAS2 | 2'-5'-oligoadenylate synthetase 2 | 5.153 | 6.74E-03 |
| ISG15 | ISG15 ubiquitin-like modifier | 4.951 | 2.10E-05 |
| OAS3 | 2'-5'-oligoadenylate synthetase 3 | 4.819 | 2.00E-05 |
| IFIT3 | interferon induced protein with tetratricopeptide repeats 3 | 4.507 | 3.25E-03 |
| USP18 | ubiquitin specific peptidase 18 | 4.151 | 6.95E-03 |
| IFITM1 | interferon induced transmembrane protein 1 | 4.084 | 1.40E-05 |
| HERC6 | HECT and RLD domain containing E3 ubiquitin protein ligase family member 6 | 4.079 | 2.43E-02 |
| TRANK1 | tetratricopeptide repeat and ankyrin repeat containing 1 | 3.788 | 1.07E-02 |
| SAMHD1 | SAM and HD domain containing deoxynucleoside triphosphate triphosphohydrolase 1 | 3.783 | 3.08E-03 |
| SLC22A18 | solute carrier family 22 member 18 | 3.772 | 4.96E-02 |
| TLDC2 | TBC/LysM-associated domain containing 2 | 3.535 | 1.84E-02 |
| STAT1 | signal transducer and activator of transcription 1 | 3.103 | 4.30E-05 |
| PARP14 | poly(ADP-ribose) polymerase family member 14 | 3.067 | 1.97E-04 |
| UBA7 | ubiquitin like modifier activating enzyme 7 | 2.919 | 2.79E-02 |
| TRIM22 | tripartite motif containing 22 | 2.837 | 7.13E-03 |
| LY6E | lymphocyte antigen 6 family member E | 2.781 | 1.03E-04 |
| PLSCR1 | phospholipid scramblase 1 | 2.775 | 1.06E-02 |
| C7orf49 | chromosome 7 open reading frame 49 | 2.656 | 1.41E-04 |
| SECTM1 | secreted and transmembrane 1 | 2.641 | 2.45E-02 |
| BST2 | bone marrow stromal cell antigen 2 | 2.57 | 7.37E-04 |
| PARP9 | poly(ADP-ribose) polymerase family member 9 | 2.485 | 6.00E-05 |
| CCL2 | C-C motif chemokine ligand 2 | 2.459 | 6.64E-04 |
| IFIT5 | interferon induced protein with tetratricopeptide repeats 5 | 2.459 | 3.39E-02 |
| RNF213 | ring finger protein 213 | 2.423 | 1.06E-04 |
| UBE2L6 | ubiquitin conjugating enzyme E2 L6 | 2.332 | 1.06E-03 |
| DTX3L | deltex E3 ubiquitin ligase 3L | 2.318 | 8.01E-03 |
| SP110 | SP110 nuclear body protein | 2.303 | 1.79E-02 |
| APOL1 | apolipoprotein L1 | 2.299 | 3.10E-03 |
| STAT2 | signal transducer and activator of transcription 2 | 2.166 | 9.38E-04 |
| PARP10 | poly(ADP-ribose) polymerase family member 10 | 2.144 | 1.97E-04 |
| PLEKHA4 | pleckstrin homology domain containing A4 | 2.044 | 1.16E-03 |
| SCD | stearoyl-CoA desaturase | 2.033 | 3.86E-02 |
| IRF1 | interferon regulatory factor 1 | 1.893 | 6.91E-03 |
| SPAG4 | sperm associated antigen 4 | 1.848 | 1.61E-02 |
| TRIM14 | tripartite motif containing 14 | 1.796 | 1.79E-02 |
| TRIM25 | tripartite motif containing 25 | 1.793 | 1.74E-03 |
| ANGPTL6 | angiopoietin like 6 | 1.785 | 2.19E-03 |
| ZNFX1 | zinc finger NFX1-type containing 1 | 1.772 | 1.97E-04 |
| IFITM3 | interferon induced transmembrane protein 3 | 1.733 | 3.76E-03 |
| AGRN | agrin | 1.731 | 6.81E-03 |
| IFI35 | interferon induced protein 35 | 1.716 | 3.52E-02 |
| C19orf66 | chromosome 19 open reading frame 66 | 1.613 | 2.47E-02 |
| GBP1 | guanylate binding protein 1 | 1.606 | 4.96E-02 |
| GSDMD | gasdermin D | 1.585 | 2.88E-02 |
| TYMP | thymidine phosphorylase | 1.555 | 1.25E-03 |
| C1R | complement C1r | 1.546 | 2.47E-02 |
| ADAR | adenosine deaminase, RNA specific | 1.512 | 9.53E-04 |
| ACO1 | aconitase 1 | 1.494 | 3.69E-02 |
| TAP1 | transporter 1, ATP binding cassette subfamily B member | 1.491 | 1.06E-02 |
| LGALS3BP | galectin 3 binding protein | 1.405 | 2.73E-02 |
| CDSN | corneodesmosin | 1.367 | 2.60E-02 |
| IRF7 | interferon regulatory factor 7 | 1.338 | 2.62E-02 |
| NLGN2 | neuroligin 2 | 1.292 | 4.11E-02 |
| TMEM132A | transmembrane protein 132A | 1.185 | 2.26E-02 |
| ADAMTSL4 | ADAMTS like 4 | 1.156 | 4.96E-02 |
| GPX3 | glutathione peroxidase 3 | 1.136 | 3.41E-02 |
| PML | promyelocytic leukemia | 1.089 | 4.96E-02 |
| OGFR | opioid growth factor receptor | 1.083 | 1.47E-02 |
| SUN2 | Sad1 and UNC84 domain containing 2 | 0.98 | 3.44E-02 |
| HIST1H2BD | histone cluster 1 H2B family member d | -1.138 | 2.79E-02 |
| TUBA1C | tubulin alpha 1c | -1.284 | 7.13E-03 |
| HIST1H3B | histone cluster 1 H3 family member b | -1.465 | 1.27E-02 |
| HIST1H1B | histone cluster 1 H1 family member b | -1.504 | 3.58E-04 |

**Supplementary Table S3.** Differentially expressed genes (p<0.05) of ZIKV-infected hSeC at 72 hpi. Ranked by Log2 FC.

| **Gene Symbol** | **Gene Name** | **Log2 FC** | **p-value** |
| --- | --- | --- | --- |
| IFI27 | interferon alpha inducible protein 27 | 7.343 | 2.90E-05 |
| IFI6 | interferon alpha inducible protein 6 | 7.131 | 1.00E-06 |
| DHX58 | DExH-box helicase 58 | 6.197 | 2.80E-05 |
| MX1 | MX dynamin like GTPase 1 | 6.108 | 1.90E-05 |
| OAS1 | 2'-5'-oligoadenylate synthetase 1 | 6.061 | 4.22E-04 |
| EPSTI1 | epithelial stromal interaction 1 | 6.045 | 1.44E-04 |
| FNDC11 | fibronectin type III domain containing 11 | 5.949 | 2.37E-04 |
| RSAD2 | radical S-adenosyl methionine domain containing 2 | 5.867 | 3.76E-03 |
| CMPK2 | cytidine/uridine monophosphate kinase 2 | 5.841 | 5.20E-05 |
| ATF3 | activating transcription factor 3 | 5.753 | 1.22E-04 |
| MX2 | MX dynamin like GTPase 2 | 5.551 | 3.32E-03 |
| OASL | 2'-5'-oligoadenylate synthetase like | 5.318 | 2.63E-03 |
| IFIH1 | interferon induced with helicase C domain 1 | 5.309 | 5.17E-04 |
| IFIT1 | interferon induced protein with tetratricopeptide repeats 1 | 5.244 | 2.05E-02 |
| IFI44L | interferon induced protein 44 like | 5.229 | 1.17E-02 |
| BATF2 | basic leucine zipper ATF-like transcription factor 2 | 5.149 | 1.63E-03 |
| OAS3 | 2'-5'-oligoadenylate synthetase 3 | 4.919 | 2.00E-06 |
| ISG15 | ISG15 ubiquitin-like modifier | 4.822 | 6.00E-06 |
| ISG20 | interferon stimulated exonuclease gene 20 | 4.696 | 2.13E-02 |
| IL4I1 | interleukin 4 induced 1 | 4.581 | 6.11E-04 |
| IFITM1 | interferon induced transmembrane protein 1 | 4.557 | 1.00E-06 |
| AATK | apoptosis associated tyrosine kinase | 4.519 | 3.01E-02 |
| OAS2 | 2'-5'-oligoadenylate synthetase 2 | 4.338 | 8.30E-03 |
| IFIT3 | interferon induced protein with tetratricopeptide repeats 3 | 4.25 | 1.90E-03 |
| HERC6 | HECT and RLD domain containing E3 ubiquitin protein ligase family member 6 | 4.12 | 5.66E-03 |
| LTB | lymphotoxin beta | 3.963 | 3.30E-02 |
| TLDC2 | TBC/LysM-associated domain containing 2 | 3.716 | 2.22E-03 |
| SAMHD1 | SAM and HD domain containing deoxynucleoside triphosphate triphosphohydrolase 1 | 3.677 | 1.23E-03 |
| SMTNL1 | smoothelin like 1 | 3.655 | 6.30E-03 |
| TRANK1 | tetratricopeptide repeat and ankyrin repeat containing 1 | 3.601 | 4.88E-03 |
| USP18 | ubiquitin specific peptidase 18 | 3.546 | 7.48E-03 |
| SLC22A18 | solute carrier family 22 member 18 | 3.521 | 1.85E-02 |
| PRICKLE3 | prickle planar cell polarity protein 3 | 3.48 | 1.63E-02 |
| LY6E | lymphocyte antigen 6 family member E | 3.418 | 1.00E-06 |
| HES4 | hes family bHLH transcription factor 4 | 3.34 | 1.25E-02 |
| UCP3 | uncoupling protein 3 | 3.318 | 1.01E-02 |
| ASB7 | ankyrin repeat and SOCS box containing 7 | 3.263 | 1.51E-02 |
| BST2 | bone marrow stromal cell antigen 2 | 3.199 | 8.00E-06 |
| C7orf49 | chromosome 7 open reading frame 49 | 2.995 | 4.00E-06 |
| CETP | cholesteryl ester transfer protein | 2.931 | 4.12E-02 |
| BCAN | brevican | 2.927 | 3.43E-04 |
| PSD | pleckstrin and Sec7 domain containing | 2.921 | 5.76E-03 |
| STAT1 | signal transducer and activator of transcription 1 | 2.91 | 1.70E-05 |
| IL7 | interleukin 7 | 2.904 | 1.12E-03 |
| TMEM102 | transmembrane protein 102 | 2.873 | 1.22E-02 |
| GDF15 | growth differentiation factor 15 | 2.824 | 2.52E-03 |
| UBA7 | ubiquitin like modifier activating enzyme 7 | 2.824 | 8.61E-03 |
| TRIM22 | tripartite motif containing 22 | 2.786 | 2.27E-03 |
| PARP14 | poly(ADP-ribose) polymerase family member 14 | 2.725 | 1.92E-04 |
| RRAD | RRAD, Ras related glycolysis inhibitor and calcium channel regulator | 2.719 | 9.87E-03 |
| NFKBIE | NFKB inhibitor epsilon | 2.69 | 3.21E-02 |
| ATP10A | ATPase phospholipid transporting 10A (putative) | 2.666 | 2.90E-02 |
| PARP9 | poly(ADP-ribose) polymerase family member 9 | 2.61 | 4.00E-06 |
| CCL2 | C-C motif chemokine ligand 2 | 2.549 | 7.00E-05 |
| IFIT5 | interferon induced protein with tetratricopeptide repeats 5 | 2.545 | 5.78E-03 |
| IRF1 | interferon regulatory factor 1 | 2.538 | 4.70E-05 |
| PLSCR1 | phospholipid scramblase 1 | 2.538 | 5.69E-03 |
| APOL1 | apolipoprotein L1 | 2.46 | 2.79E-04 |
| MASP1 | mannan binding lectin serine peptidase 1 | 2.457 | 3.12E-02 |
| IL23A | interleukin 23 subunit alpha | 2.454 | 1.30E-02 |
| MYO15B | myosin XVB | 2.436 | 2.41E-02 |
| POLM | DNA polymerase mu | 2.412 | 4.64E-02 |
| NICN1 | nicolin 1 | 2.404 | 4.91E-03 |
| GMPR | guanosine monophosphate reductase | 2.386 | 4.23E-02 |
| CA9 | carbonic anhydrase 9 | 2.362 | 3.65E-02 |
| PRRT2 | proline rich transmembrane protein 2 | 2.349 | 2.19E-02 |
| PKD1L1 | polycystin 1 like 1, transient receptor potential channel interacting | 2.341 | 4.62E-02 |
| CCDC88B | coiled-coil domain containing 88B | 2.336 | 4.92E-02 |
| VWA1 | von Willebrand factor A domain containing 1 | 2.329 | 3.39E-02 |
| ADAMTS13 | ADAM metallopeptidase with thrombospondin type 1 motif 13 | 2.328 | 3.01E-02 |
| SECTM1 | secreted and transmembrane 1 | 2.327 | 1.39E-02 |
| C1R | complement C1r | 2.326 | 4.40E-05 |
| C3orf58 | chromosome 3 open reading frame 58 | 2.304 | 3.10E-02 |
| DTX3L | deltex E3 ubiquitin ligase 3L | 2.283 | 2.07E-03 |
| SPAG4 | sperm associated antigen 4 | 2.258 | 3.09E-04 |
| ZNF862 | zinc finger protein 862 | 2.197 | 6.30E-03 |
| RNF213 | ring finger protein 213 | 2.146 | 6.60E-05 |
| SCD | stearoyl-CoA desaturase | 2.113 | 5.61E-03 |
| AGRN | agrin | 2.092 | 1.07E-04 |
| PARP10 | poly(ADP-ribose) polymerase family member 10 | 2.08 | 4.40E-05 |
| FBXL17 | F-box and leucine rich repeat protein 17 | 2.074 | 4.85E-02 |
| PSORS1C2 | psoriasis susceptibility 1 candidate 2 | 2.064 | 2.19E-02 |
| HCG4 | HLA complex group 4 (non-protein coding) | 2.056 | 4.00E-06 |
| PLEKHA4 | pleckstrin homology domain containing A4 | 2.055 | 1.73E-04 |
| UBE2L6 | ubiquitin conjugating enzyme E2 L6 | 2.049 | 9.15E-04 |
| ADM | adrenomedullin | 2.047 | 2.64E-02 |
| IFITM3 | interferon induced transmembrane protein 3 | 2.039 | 8.00E-05 |
| DDIT4 | DNA damage inducible transcript 4 | 2.039 | 3.47E-03 |
| ELMO3 | engulfment and cell motility 3 | 2.037 | 3.21E-02 |
| IL6 | interleukin 6 | 2.004 | 1.28E-02 |
| HLA-B | major histocompatibility complex, class I, B | 1.976 | 2.80E-05 |
| TAP1 | transporter 1, ATP binding cassette subfamily B member | 1.966 | 7.40E-05 |
| OSCAR | osteoclast associated, immunoglobulin-like receptor | 1.959 | 2.58E-04 |
| STAT2 | signal transducer and activator of transcription 2 | 1.915 | 6.35E-04 |
| SP110 | SP110 nuclear body protein | 1.912 | 1.51E-02 |
| TSSK3 | testis specific serine kinase 3 | 1.908 | 9.15E-04 |
| PHF21A | PHD finger protein 21A | 1.907 | 3.09E-02 |
| CARD14 | caspase recruitment domain family member 14 | 1.905 | 8.86E-04 |
| CD82 | CD82 molecule | 1.904 | 2.51E-02 |
| ZSWIM5 | zinc finger SWIM-type containing 5 | 1.898 | 7.48E-03 |
| ANGPTL6 | angiopoietin like 6 | 1.893 | 1.61E-04 |
| CACFD1 | calcium channel flower domain containing 1 | 1.887 | 6.30E-03 |
| LGALS3BP | galectin 3 binding protein | 1.884 | 1.92E-04 |
| ESM1 | endothelial cell specific molecule 1 | 1.869 | 4.50E-02 |
| CLSTN3 | calsyntenin 3 | 1.86 | 4.52E-02 |
| IFI35 | interferon induced protein 35 | 1.838 | 4.05E-03 |
| SOD2 | superoxide dismutase 2 | 1.82 | 8.03E-04 |
| RASD1 | ras related dexamethasone induced 1 | 1.812 | 8.60E-03 |
| KAT2A | lysine acetyltransferase 2A | 1.809 | 1.25E-02 |
| BDKRB2 | bradykinin receptor B2 | 1.799 | 1.23E-02 |
| BTG1 | BTG anti-proliferation factor 1 | 1.796 | 4.82E-02 |
| HLA-F | major histocompatibility complex, class I, F | 1.793 | 2.69E-04 |
| WDR73 | WD repeat domain 73 | 1.791 | 3.35E-03 |
| CRACR2B | calcium release activated channel regulator 2B | 1.791 | 3.13E-02 |
| ODF3B | outer dense fiber of sperm tails 3B | 1.79 | 1.62E-03 |
| HLA-C | major histocompatibility complex, class I, C | 1.783 | 1.92E-04 |
| TYMP | thymidine phosphorylase | 1.77 | 4.40E-05 |
| HLA-G | major histocompatibility complex, class I, G | 1.757 | 5.25E-03 |
| PARP12 | poly(ADP-ribose) polymerase family member 12 | 1.749 | 2.43E-02 |
| C1S | complement C1s | 1.742 | 3.12E-04 |
| SAMD9L | sterile alpha motif domain containing 9 like | 1.738 | 3.37E-02 |
| ENO2 | enolase 2 | 1.733 | 7.71E-04 |
| TMEM132A | transmembrane protein 132A | 1.704 | 6.00E-05 |
| CFH | complement factor H | 1.678 | 6.54E-03 |
| ARID5A | AT-rich interaction domain 5A | 1.668 | 2.19E-02 |
| NAGS | N-acetylglutamate synthase | 1.659 | 2.07E-03 |
| C19orf66 | chromosome 19 open reading frame 66 | 1.659 | 3.32E-03 |
| DYRK1B | dual specificity tyrosine phosphorylation regulated kinase 1B | 1.654 | 4.10E-02 |
| GP1BA | glycoprotein Ib platelet alpha subunit | 1.638 | 1.05E-02 |
| MZF1 | myeloid zinc finger 1 | 1.629 | 2.14E-04 |
| CFB | complement factor B | 1.622 | 1.39E-02 |
| APOL6 | apolipoprotein L6 | 1.617 | 4.21E-02 |
| ZNFX1 | zinc finger NFX1-type containing 1 | 1.611 | 8.00E-05 |
| MAML3 | mastermind like transcriptional coactivator 3 | 1.602 | 1.70E-02 |
| AKR1B1 | aldo-keto reductase family 1 member B | 1.597 | 8.94E-03 |
| CADM4 | cell adhesion molecule 4 | 1.594 | 2.19E-02 |
| RNF24 | ring finger protein 24 | 1.589 | 3.65E-02 |
| PDGFRB | platelet derived growth factor receptor beta | 1.588 | 7.76E-03 |
| MDK | midkine (neurite growth-promoting factor 2) | 1.586 | 4.05E-03 |
| C1RL | complement C1r subcomponent like | 1.583 | 8.95E-03 |
| HLA-A | major histocompatibility complex, class I, A | 1.571 | 1.68E-03 |
| MC1R | melanocortin 1 receptor | 1.567 | 4.43E-02 |
| ACO1 | aconitase 1 | 1.565 | 4.16E-03 |
| ACAP1 | ArfGAP with coiled-coil, ankyrin repeat and PH domains 1 | 1.561 | 7.26E-03 |
| ITGB7 | integrin subunit beta 7 | 1.54 | 4.39E-02 |
| NFE2L3 | nuclear factor, erythroid 2 like 3 | 1.529 | 2.26E-02 |
| MTERF4 | mitochondrial transcription termination factor 4 | 1.523 | 4.04E-02 |
| PSMB10 | proteasome subunit beta 10 | 1.52 | 2.01E-02 |
| COL7A1 | collagen type VII alpha 1 chain | 1.518 | 9.20E-03 |
| IFITM2 | interferon induced transmembrane protein 2 | 1.507 | 7.51E-03 |
| CXCL3 | C-X-C motif chemokine ligand 3 | 1.492 | 7.26E-03 |
| GSDMD | gasdermin D | 1.492 | 8.99E-03 |
| C3 | complement C3 | 1.488 | 4.05E-03 |
| ADGRA3 | adhesion G protein-coupled receptor A3 | 1.485 | 1.25E-02 |
| SAMD14 | sterile alpha motif domain containing 14 | 1.481 | 2.83E-02 |
| CDSN | corneodesmosin | 1.48 | 2.02E-03 |
| SH2D3A | SH2 domain containing 3A | 1.471 | 1.94E-02 |
| IRF9 | interferon regulatory factor 9 | 1.47 | 1.39E-02 |
| HLA-J | major histocompatibility complex, class I, J (pseudogene) | 1.468 | 9.58E-03 |
| PDK1 | pyruvate dehydrogenase kinase 1 | 1.465 | 4.23E-02 |
| GPT | glutamic--pyruvic transaminase | 1.456 | 1.28E-02 |
| KLF4 | Kruppel like factor 4 | 1.454 | 4.39E-02 |
| NTN3 | netrin 3 | 1.453 | 7.48E-03 |
| MAMDC4 | MAM domain containing 4 | 1.446 | 3.43E-04 |
| CD68 | CD68 molecule | 1.437 | 1.30E-03 |
| ADAR | adenosine deaminase, RNA specific | 1.431 | 2.28E-04 |
| CSF3 | colony stimulating factor 3 | 1.424 | 2.74E-03 |
| MFSD13A | major facilitator superfamily domain containing 13A | 1.424 | 4.64E-02 |
| PTN | pleiotrophin | 1.421 | 1.33E-02 |
| TRIM14 | tripartite motif containing 14 | 1.416 | 1.92E-02 |
| GPX3 | glutathione peroxidase 3 | 1.41 | 6.52E-04 |
| BBC3 | BCL2 binding component 3 | 1.408 | 2.52E-02 |
| SLC6A6 | solute carrier family 6 member 6 | 1.407 | 4.90E-03 |
| INSIG1 | insulin induced gene 1 | 1.4 | 1.72E-02 |
| TMEM158 | transmembrane protein 158 (gene/pseudogene) | 1.39 | 1.22E-02 |
| TBC1D22B | TBC1 domain family member 22B | 1.39 | 2.22E-02 |
| ANKZF1 | ankyrin repeat and zinc finger domain containing 1 | 1.387 | 1.30E-02 |
| ANGPTL2 | angiopoietin like 2 | 1.381 | 8.71E-03 |
| IFI16 | interferon gamma inducible protein 16 | 1.378 | 1.57E-02 |
| USP6NL | USP6 N-terminal like | 1.377 | 2.01E-02 |
| DHRS3 | dehydrogenase/reductase 3 | 1.377 | 3.65E-02 |
| CD37 | CD37 molecule | 1.374 | 3.44E-02 |
| CHAD | chondroadherin | 1.368 | 1.28E-02 |
| AMT | aminomethyltransferase | 1.367 | 3.74E-02 |
| ADAMTSL4 | ADAMTS like 4 | 1.361 | 1.95E-03 |
| TBC1D17 | TBC1 domain family member 17 | 1.36 | 8.61E-03 |
| ABCC1 | ATP binding cassette subfamily C member 1 | 1.341 | 7.32E-03 |
| EMC9 | ER membrane protein complex subunit 9 | 1.336 | 3.94E-02 |
| B2M | beta-2-microglobulin | 1.33 | 1.51E-02 |
| ZNF205 | zinc finger protein 205 | 1.324 | 3.71E-02 |
| H1F0 | H1 histone family member 0 | 1.311 | 8.60E-05 |
| FTH1 | ferritin heavy chain 1 | 1.298 | 3.89E-03 |
| KLF9 | Kruppel like factor 9 | 1.297 | 3.78E-03 |
| TNFRSF6B | TNF receptor superfamily member 6b | 1.293 | 4.21E-02 |
| NLGN2 | neuroligin 2 | 1.287 | 7.09E-03 |
| TRIM25 | tripartite motif containing 25 | 1.284 | 7.15E-03 |
| CHKA | choline kinase alpha | 1.276 | 8.99E-03 |
| CEBPD | CCAAT/enhancer binding protein delta | 1.268 | 1.26E-03 |
| LRRC14 | leucine rich repeat containing 14 | 1.263 | 7.38E-03 |
| CRELD1 | cysteine rich with EGF like domains 1 | 1.255 | 2.45E-02 |
| TNFAIP2 | TNF alpha induced protein 2 | 1.239 | 4.53E-02 |
| HLA-E | major histocompatibility complex, class I, E | 1.231 | 1.24E-02 |
| PID1 | phosphotyrosine interaction domain containing 1 | 1.228 | 2.25E-03 |
| TNIP1 | TNFAIP3 interacting protein 1 | 1.225 | 3.32E-03 |
| LIPG | lipase G, endothelial type | 1.225 | 2.92E-02 |
| FADS1 | fatty acid desaturase 1 | 1.205 | 1.03E-02 |
| ZFPM1 | zinc finger protein, FOG family member 1 | 1.201 | 1.58E-02 |
| PIK3IP1 | phosphoinositide-3-kinase interacting protein 1 | 1.196 | 1.00E-02 |
| NFKBIZ | NFKB inhibitor zeta | 1.195 | 1.29E-02 |
| C1QTNF1 | C1q and tumor necrosis factor related protein 1 | 1.193 | 1.42E-02 |
| RUSC1 | RUN and SH3 domain containing 1 | 1.19 | 1.15E-02 |
| ICAM5 | intercellular adhesion molecule 5 | 1.188 | 3.13E-02 |
| AHR | aryl hydrocarbon receptor | 1.187 | 7.32E-03 |
| CUL9 | cullin 9 | 1.186 | 4.50E-02 |
| CEBPB | CCAAT/enhancer binding protein beta | 1.185 | 1.08E-03 |
| PSME1 | proteasome activator subunit 1 | 1.185 | 5.18E-03 |
| CXCL2 | C-X-C motif chemokine ligand 2 | 1.185 | 2.26E-02 |
| VEGFA | vascular endothelial growth factor A | 1.181 | 9.26E-03 |
| FADS2 | fatty acid desaturase 2 | 1.179 | 3.47E-02 |
| SLC27A1 | solute carrier family 27 member 1 | 1.172 | 2.68E-03 |
| CSPG4 | chondroitin sulfate proteoglycan 4 | 1.17 | 4.97E-02 |
| PSME2 | proteasome activator subunit 2 | 1.168 | 4.99E-02 |
| FAM160B2 | family with sequence similarity 160 member B2 | 1.165 | 9.42E-03 |
| NFKB1 | nuclear factor kappa B subunit 1 | 1.161 | 2.44E-02 |
| TAF9B | TATA-box binding protein associated factor 9b | 1.154 | 4.37E-02 |
| SOX12 | SRY-box 12 | 1.153 | 2.88E-03 |
| HIP1R | huntingtin interacting protein 1 related | 1.151 | 2.60E-02 |
| RBP5 | retinol binding protein 5 | 1.151 | 4.92E-02 |
| PLEKHG4 | pleckstrin homology and RhoGEF domain containing G4 | 1.149 | 1.35E-02 |
| PGAM2 | phosphoglycerate mutase 2 | 1.136 | 4.68E-03 |
| EVA1B | eva-1 homolog B | 1.134 | 1.85E-02 |
| NAMPT | nicotinamide phosphoribosyltransferase | 1.128 | 4.45E-02 |
| ARRDC1 | arrestin domain containing 1 | 1.125 | 3.32E-03 |
| KDM4B | lysine demethylase 4B | 1.122 | 4.69E-02 |
| TNFAIP3 | TNF alpha induced protein 3 | 1.119 | 8.61E-03 |
| NANS | N-acetylneuraminate synthase | 1.119 | 3.73E-02 |
| MRPL43 | mitochondrial ribosomal protein L43 | 1.115 | 2.76E-02 |
| OGFR | opioid growth factor receptor | 1.111 | 1.51E-03 |
| EGR1 | early growth response 1 | 1.109 | 4.21E-02 |
| PNRC1 | proline rich nuclear receptor coactivator 1 | 1.106 | 4.92E-02 |
| FAAP100 | Fanconi anemia core complex associated protein 100 | 1.104 | 2.56E-02 |
| SEC14L2 | SEC14 like lipid binding 2 | 1.096 | 2.80E-02 |
| OSGIN2 | oxidative stress induced growth inhibitor family member 2 | 1.091 | 3.57E-02 |
| AMIGO3 | adhesion molecule with Ig like domain 3 | 1.085 | 2.45E-02 |
| SYTL4 | synaptotagmin like 4 | 1.077 | 1.27E-02 |
| TCTEX1D4 | Tctex1 domain containing 4 | 1.072 | 7.98E-03 |
| FUT11 | fucosyltransferase 11 | 1.07 | 4.98E-02 |
| FAM219A | family with sequence similarity 219 member A | 1.069 | 3.80E-02 |
| NFKBIA | NFKB inhibitor alpha | 1.065 | 5.12E-03 |
| STC1 | stanniocalcin 1 | 1.05 | 1.57E-02 |
| PGD | phosphogluconate dehydrogenase | 1.049 | 3.78E-02 |
| PHPT1 | phosphohistidine phosphatase 1 | 1.043 | 1.63E-02 |
| ST3GAL1 | ST3 beta-galactoside alpha-2,3-sialyltransferase 1 | 1.037 | 4.97E-02 |
| CSF1 | colony stimulating factor 1 | 1.024 | 1.51E-02 |
| TNC | tenascin C | 1.024 | 2.97E-02 |
| SCARB2 | scavenger receptor class B member 2 | 1.023 | 1.05E-02 |
| JUND | JunD proto-oncogene, AP-1 transcription factor subunit | 1.017 | 1.98E-02 |
| CCDC152 | coiled-coil domain containing 152 | 1.007 | 4.82E-02 |
| BHLHE41 | basic helix-loop-helix family member e41 | 1.006 | 3.58E-02 |
| NRM | nurim (nuclear envelope membrane protein) | 1.003 | 3.76E-02 |
| IRF7 | interferon regulatory factor 7 | 0.998 | 3.18E-02 |
| SLC39A14 | solute carrier family 39 member 14 | 0.991 | 3.91E-02 |
| MYH7B | myosin heavy chain 7B | 0.99 | 1.22E-02 |
| TMEM80 | transmembrane protein 80 | 0.986 | 3.54E-02 |
| PML | promyelocytic leukemia | 0.954 | 1.80E-02 |
| ZC3H12A | zinc finger CCCH-type containing 12A | 0.95 | 1.62E-02 |
| KLHL17 | kelch like family member 17 | 0.945 | 4.69E-02 |
| CXCL1 | C-X-C motif chemokine ligand 1 | 0.942 | 8.88E-03 |
| SYT15 | synaptotagmin 15 | 0.94 | 4.80E-02 |
| SLC22A23 | solute carrier family 22 member 23 | 0.936 | 1.22E-02 |
| WTAP | Wilms tumor 1 associated protein | 0.936 | 3.63E-02 |
| AP1G2 | adaptor related protein complex 1 gamma 2 subunit | 0.932 | 1.28E-02 |
| C21orf2 | chromosome 21 open reading frame 2 | 0.926 | 4.88E-03 |
| SUN2 | Sad1 and UNC84 domain containing 2 | 0.915 | 8.60E-03 |
| LOXL4 | lysyl oxidase like 4 | 0.899 | 4.26E-02 |
| LAMB1 | laminin subunit beta 1 | 0.891 | 3.54E-02 |
| DNAH10 | dynein axonemal heavy chain 10 | 0.886 | 1.71E-02 |
| TNFRSF10B | TNF receptor superfamily member 10b | 0.883 | 4.82E-02 |
| CXCL8 | C-X-C motif chemokine ligand 8 | 0.868 | 2.44E-02 |
| TMEM120B | transmembrane protein 120B | 0.867 | 4.28E-02 |
| CDC42EP3 | CDC42 effector protein 3 | 0.823 | 4.30E-02 |
| ZNF219 | zinc finger protein 219 | 0.804 | 4.80E-02 |
| C19orf71 | chromosome 19 open reading frame 71 | 0.794 | 6.30E-03 |
| JUNB | JunB proto-oncogene, AP-1 transcription factor subunit | 0.788 | 4.58E-02 |
| CD27 | CD27 molecule | 0.782 | 4.16E-02 |
| MRNIP | MRN complex interacting protein | 0.777 | 8.61E-03 |
| OBSCN | obscurin, cytoskeletal calmodulin and titin-interacting RhoGEF | 0.757 | 4.11E-02 |
| APC2 | APC2, WNT signaling pathway regulator | 0.673 | 2.38E-02 |
| MMP2 | matrix metallopeptidase 2 | 0.639 | 3.51E-02 |
| FCHSD1 | FCH and double SH3 domains 1 | 0.638 | 4.10E-02 |
| MSC | musculin | 0.608 | 3.85E-02 |
| H1FX | H1 histone family member X | 0.574 | 4.21E-02 |
| SARM1 | sterile alpha and TIR motif containing 1 | -0.59 | 4.29E-02 |
| ARHGEF39 | Rho guanine nucleotide exchange factor 39 | -0.607 | 2.88E-02 |
| MBNL1 | muscleblind like splicing regulator 1 | -0.651 | 4.58E-02 |
| VIM | vimentin | -0.679 | 4.82E-02 |
| DRAP1 | DR1 associated protein 1 | -0.713 | 4.69E-02 |
| POLG | DNA polymerase gamma, catalytic subunit | -0.714 | 3.09E-02 |
| LRTOMT | leucine rich transmembrane and O-methyltransferase domain containing | -0.715 | 2.70E-02 |
| OPA3 | OPA3, outer mitochondrial membrane lipid metabolism regulator | -0.723 | 4.30E-02 |
| MYOF | myoferlin | -0.744 | 4.62E-02 |
| HIST1H1E | histone cluster 1 H1 family member e | -0.746 | 3.65E-02 |
| UBE2S | ubiquitin conjugating enzyme E2 S | -0.752 | 2.25E-02 |
| KANK2 | KN motif and ankyrin repeat domains 2 | -0.764 | 4.43E-02 |
| ACTB | actin beta | -0.77 | 3.04E-02 |
| LRRC59 | leucine rich repeat containing 59 | -0.784 | 2.74E-02 |
| SAMD11 | sterile alpha motif domain containing 11 | -0.785 | 3.85E-02 |
| LMNB2 | lamin B2 | -0.798 | 1.70E-02 |
| DNMT1 | DNA methyltransferase 1 | -0.801 | 8.08E-03 |
| LMNA | lamin A/C | -0.801 | 2.33E-02 |
| HSP90AA1 | heat shock protein 90 alpha family class A member 1 | -0.803 | 8.18E-03 |
| KPNB1 | karyopherin subunit beta 1 | -0.83 | 1.39E-02 |
| FAT1 | FAT atypical cadherin 1 | -0.834 | 3.69E-02 |
| ACTG1 | actin gamma 1 | -0.84 | 1.51E-02 |
| FUS | FUS RNA binding protein | -0.849 | 4.23E-02 |
| RPL7A | ribosomal protein L7a | -0.852 | 2.33E-02 |
| CNN2 | calponin 2 | -0.857 | 3.58E-02 |
| ATP5F1 | ATP synthase, H+ transporting, mitochondrial Fo complex subunit B1 | -0.865 | 9.62E-03 |
| MXD3 | MAX dimerization protein 3 | -0.865 | 3.03E-02 |
| CPT1A | carnitine palmitoyltransferase 1A | -0.873 | 2.64E-02 |
| C6orf201 | chromosome 6 open reading frame 201 | -0.873 | 3.15E-02 |
| SERBP1 | SERPINE1 mRNA binding protein 1 | -0.877 | 1.02E-02 |
| DOC2A | double C2 domain alpha | -0.881 | 4.86E-02 |
| CHAF1A | chromatin assembly factor 1 subunit A | -0.889 | 4.13E-02 |
| NF2 | neurofibromin 2 | -0.898 | 4.85E-02 |
| SPNS2 | sphingolipid transporter 2 | -0.909 | 2.28E-03 |
| EIF4E2 | eukaryotic translation initiation factor 4E family member 2 | -0.912 | 4.45E-02 |
| UACA | uveal autoantigen with coiled-coil domains and ankyrin repeats | -0.929 | 2.60E-02 |
| FAM151A | family with sequence similarity 151 member A | -0.937 | 3.91E-02 |
| ENDOG | endonuclease G | -0.939 | 3.02E-02 |
| PLS3 | plastin 3 | -0.94 | 2.17E-02 |
| TYMSOS | TYMS opposite strand | -0.941 | 3.30E-02 |
| LNP1 | leukemia NUP98 fusion partner 1 | -0.941 | 3.65E-02 |
| HSPA8 | heat shock protein family A (Hsp70) member 8 | -0.942 | 1.34E-02 |
| HNRNPR | heterogeneous nuclear ribonucleoprotein R | -0.97 | 1.94E-02 |
| TACC3 | transforming acidic coiled-coil containing protein 3 | -0.975 | 2.81E-02 |
| ANXA2 | annexin A2 | -0.978 | 1.87E-02 |
| AHNAK | AHNAK nucleoprotein | -0.985 | 9.51E-03 |
| MRPL57 | mitochondrial ribosomal protein L57 | -0.985 | 4.87E-02 |
| SZT2 | seizure threshold 2 homolog (mouse) | -0.992 | 4.80E-04 |
| INCENP | inner centromere protein | -0.993 | 5.61E-03 |
| HIST1H2AC | histone cluster 1 H2A family member c | -1 | 4.04E-02 |
| H2AFX | H2A histone family member X | -1.004 | 4.90E-03 |
| ADCY3 | adenylate cyclase 3 | -1.009 | 1.04E-02 |
| SSR3 | signal sequence receptor subunit 3 | -1.019 | 1.51E-02 |
| EIF2AK1 | eukaryotic translation initiation factor 2 alpha kinase 1 | -1.034 | 3.02E-02 |
| TPX2 | TPX2, microtubule nucleation factor | -1.036 | 2.59E-02 |
| FLNC | filamin C | -1.044 | 3.00E-02 |
| RRP12 | ribosomal RNA processing 12 homolog | -1.045 | 3.29E-02 |
| SUGT1P3 | SGT1 homolog, MIS12 kinetochore complex assembly cochaperone pseudogene 3 | -1.046 | 3.65E-02 |
| LASP1 | LIM and SH3 protein 1 | -1.053 | 9.15E-03 |
| PCSK7 | proprotein convertase subtilisin/kexin type 7 | -1.058 | 4.58E-02 |
| PAQR5 | progestin and adipoQ receptor family member 5 | -1.059 | 3.74E-02 |
| SDC1 | syndecan 1 | -1.073 | 3.30E-02 |
| ALYREF | Aly/REF export factor | -1.075 | 7.75E-03 |
| HIST1H1C | histone cluster 1 H1 family member c | -1.076 | 1.24E-03 |
| TUBB2A | tubulin beta 2A class IIa | -1.09 | 3.74E-02 |
| GRWD1 | glutamate rich WD repeat containing 1 | -1.094 | 4.45E-02 |
| GNL2 | G protein nucleolar 2 | -1.096 | 2.45E-02 |
| ADAM19 | ADAM metallopeptidase domain 19 | -1.106 | 2.71E-02 |
| MKI67 | marker of proliferation Ki-67 | -1.107 | 1.39E-02 |
| HIST1H2BK | histone cluster 1 H2B family member k | -1.118 | 4.90E-03 |
| OPN1SW | opsin 1, short wave sensitive | -1.121 | 1.02E-02 |
| POSTN | periostin | -1.121 | 3.65E-02 |
| PARP1 | poly(ADP-ribose) polymerase 1 | -1.142 | 1.04E-02 |
| ATAD3B | ATPase family, AAA domain containing 3B | -1.142 | 2.26E-02 |
| PODXL | podocalyxin like | -1.15 | 2.79E-02 |
| CALM1 | calmodulin 1 | -1.159 | 1.25E-02 |
| GPR39 | G protein-coupled receptor 39 | -1.166 | 8.27E-03 |
| MRAP | melanocortin 2 receptor accessory protein | -1.166 | 2.53E-02 |
| PKMYT1 | protein kinase, membrane associated tyrosine/threonine 1 | -1.167 | 4.88E-03 |
| EIF3CL | eukaryotic translation initiation factor 3 subunit C-like | -1.168 | 3.85E-02 |
| URB1 | URB1 ribosome biogenesis 1 homolog (S. cerevisiae) | -1.183 | 1.23E-02 |
| GPSM2 | G-protein signaling modulator 2 | -1.184 | 4.02E-02 |
| RRS1 | ribosome biogenesis regulator homolog | -1.186 | 2.30E-02 |
| ACTR2 | ARP2 actin related protein 2 homolog | -1.198 | 5.12E-03 |
| LDHB | lactate dehydrogenase B | -1.207 | 2.44E-02 |
| RFXANK | regulatory factor X associated ankyrin containing protein | -1.211 | 4.88E-03 |
| CMC2 | C-X9-C motif containing 2 | -1.219 | 5.50E-03 |
| CBX5 | chromobox 5 | -1.23 | 1.51E-02 |
| HIST1H2AE | histone cluster 1 H2A family member e | -1.23 | 3.42E-02 |
| KIF23 | kinesin family member 23 | -1.237 | 4.87E-02 |
| CERK | ceramide kinase | -1.238 | 6.77E-03 |
| MCM4 | minichromosome maintenance complex component 4 | -1.247 | 3.18E-02 |
| EBNA1BP2 | EBNA1 binding protein 2 | -1.247 | 3.39E-02 |
| SRM | spermidine synthase | -1.248 | 1.36E-02 |
| TIMM50 | translocase of inner mitochondrial membrane 50 | -1.253 | 4.92E-02 |
| CS | citrate synthase | -1.258 | 3.87E-02 |
| HIST1H2BN | histone cluster 1 H2B family member n | -1.261 | 2.45E-02 |
| DNASE1 | deoxyribonuclease 1 | -1.269 | 1.79E-02 |
| KIFC1 | kinesin family member C1 | -1.269 | 3.65E-02 |
| XYLT1 | xylosyltransferase 1 | -1.299 | 1.62E-02 |
| HIST1H3D | histone cluster 1 H3 family member d | -1.311 | 7.38E-03 |
| TUBB4B | tubulin beta 4B class IVb | -1.315 | 3.94E-04 |
| KIF18B | kinesin family member 18B | -1.322 | 3.87E-02 |
| HIST1H3I | histone cluster 1 H3 family member i | -1.324 | 7.98E-03 |
| HIST1H2AI | histone cluster 1 H2A family member i | -1.324 | 1.09E-02 |
| ALDH6A1 | aldehyde dehydrogenase 6 family member A1 | -1.336 | 4.45E-02 |
| TUBB6 | tubulin beta 6 class V | -1.345 | 3.78E-03 |
| HIST1H2BI | histone cluster 1 H2B family member i | -1.352 | 1.89E-02 |
| LSMEM2 | leucine rich single-pass membrane protein 2 | -1.358 | 4.69E-02 |
| ID3 | inhibitor of DNA binding 3, HLH protein | -1.361 | 2.31E-03 |
| ZNF185 | zinc finger protein 185 (LIM domain) | -1.367 | 2.47E-02 |
| KPNA2 | karyopherin subunit alpha 2 | -1.373 | 5.33E-03 |
| PAQR4 | progestin and adipoQ receptor family member 4 | -1.377 | 4.64E-02 |
| MCM5 | minichromosome maintenance complex component 5 | -1.382 | 7.09E-03 |
| POLE | DNA polymerase epsilon, catalytic subunit | -1.383 | 3.78E-02 |
| HIST1H2AH | histone cluster 1 H2A family member h | -1.388 | 3.59E-02 |
| TUBB | tubulin beta class I | -1.398 | 4.13E-04 |
| HIST2H3D | histone cluster 2 H3 family member d | -1.403 | 1.07E-02 |
| HIST1H2BC | histone cluster 1 H2B family member c | -1.404 | 4.03E-04 |
| DNAJC9 | DnaJ heat shock protein family (Hsp40) member C9 | -1.427 | 4.50E-02 |
| PLK1 | polo like kinase 1 | -1.436 | 3.42E-02 |
| HIST1H2AG | histone cluster 1 H2A family member g | -1.442 | 1.02E-02 |
| EIF3L | eukaryotic translation initiation factor 3 subunit L | -1.449 | 1.08E-02 |
| SEMA5A | semaphorin 5A | -1.462 | 3.57E-02 |
| CYCS | cytochrome c, somatic | -1.474 | 3.81E-02 |
| HIST2H3A | histone cluster 2 H3 family member a | -1.476 | 9.50E-03 |
| STAB1 | stabilin 1 | -1.49 | 8.67E-03 |
| TCF19 | transcription factor 19 | -1.493 | 6.54E-03 |
| HIST1H2BD | histone cluster 1 H2B family member d | -1.526 | 1.92E-04 |
| EME1 | essential meiotic structure-specific endonuclease 1 | -1.527 | 2.12E-02 |
| TEAD4 | TEA domain transcription factor 4 | -1.559 | 4.21E-02 |
| UBE2C | ubiquitin conjugating enzyme E2 C | -1.562 | 6.17E-03 |
| UHRF1 | ubiquitin like with PHD and ring finger domains 1 | -1.566 | 7.71E-04 |
| HIST1H3G | histone cluster 1 H3 family member g | -1.566 | 1.95E-03 |
| FAM166A | family with sequence similarity 166 member A | -1.583 | 3.06E-03 |
| TUBB2B | tubulin beta 2B class IIb | -1.594 | 7.34E-03 |
| TUBA1A | tubulin alpha 1a | -1.641 | 1.73E-04 |
| SAPCD2 | suppressor APC domain containing 2 | -1.646 | 4.41E-02 |
| HIST1H2AJ | histone cluster 1 H2A family member j | -1.662 | 2.52E-03 |
| HIST1H2BM | histone cluster 1 H2B family member m | -1.666 | 1.51E-02 |
| TUBB4A | tubulin beta 4A class IVa | -1.71 | 7.76E-03 |
| GTSE1 | G2 and S-phase expressed 1 | -1.713 | 8.60E-03 |
| CSE1L | chromosome segregation 1 like | -1.737 | 4.50E-02 |
| HIST1H4D | histone cluster 1 H4 family member d | -1.765 | 6.46E-03 |
| TUBA1B | tubulin alpha 1b | -1.778 | 1.90E-05 |
| KIAA0895 | KIAA0895 | -1.788 | 2.51E-02 |
| ARHGAP11A | Rho GTPase activating protein 11A | -1.807 | 4.94E-02 |
| OXTR | oxytocin receptor | -1.859 | 3.78E-03 |
| TUBB3 | tubulin beta 3 class III | -1.867 | 8.70E-05 |
| HIST1H3B | histone cluster 1 H3 family member b | -1.951 | 7.00E-05 |
| HIST1H3J | histone cluster 1 H3 family member j | -1.966 | 7.32E-03 |
| TUBA1C | tubulin alpha 1c | -1.971 | 6.00E-06 |
| RBM3 | RNA binding motif (RNP1, RRM) protein 3 | -1.977 | 2.79E-04 |
| GNAT2 | G protein subunit alpha transducin 2 | -1.991 | 1.05E-02 |
| HIST1H1A | histone cluster 1 H1 family member a | -2.06 | 3.80E-02 |
| ID1 | inhibitor of DNA binding 1, HLH protein | -2.065 | 1.47E-03 |
| HIST1H1B | histone cluster 1 H1 family member b | -2.084 | 1.00E-06 |
| CKAP2L | cytoskeleton associated protein 2 like | -2.102 | 1.42E-02 |
| HIST1H4C | histone cluster 1 H4 family member c | -2.198 | 2.24E-03 |
| RRP9 | ribosomal RNA processing 9, small subunit (SSU) processome component, homolog (yeast) | -2.227 | 1.34E-02 |
| ANLN | anillin actin binding protein | -2.262 | 6.01E-04 |
| OXCT1 | 3-oxoacid CoA-transferase 1 | -2.383 | 2.42E-02 |
| HIST1H4L | histone cluster 1 H4 family member l | -2.462 | 3.49E-03 |

**Supplementary Table S4.** Primers used for RT-PCR. Forward and Reverse.

| **Gene Symbol** | **Primer Sequence** |
| --- | --- |
| IFIH1 | 5’-GCTCAGAAAGCAATGCAGAGAT-3’  5’-TGGCTGAACTGTGGTTGAAAG-3’ |
| DHX58 | 5’-TTCTGCAGATGGCACTGACC-3’  5’-CACGATCAGGGAGAAGACAGT-3’ |
| DDX58 | 5’-ACCAATTAGAGCTTGCTTTGCC-3’  5’-CCACAACCTGTAGGAGCACA-3’ |
| MXA | 5’-AGTATGGTGTCGACATACCGGA-3’  5’-GAGTCTGGTAAACAGCCGAAT-3’ |
| TLR3 | 5’-GCCGTCTATTTGCCACACAC-3’  5’-CATGATTCTGTTGGATGACTGCT-3’ |
| IFNA | 5’-CTCCTTTCTCCTGCCTGAAG-3’  5’-AAGTGTCTCATCCCAAGTAGC-3’ |
| IFNB | 5’-CTCTCCTGTTGTGCTTCTCC-3’  5’-GTCAAAGTTCATCCTGTCCTTG-3’ |
| IFIT1 | 5’-TCAGGTCAAGGATAGTCT-3’  5’-TGTATTTGGTGTCTAGGAAT-3’ |
| TNFA | 5’-CCCAGGGACCTCTCTCTAATC-3’  5’-ATGGGCTACAGGCTTGTCACT-3’ |
| IL6 | 5’-ATCCAGTTGCCTTCTTGGGACTGA-3’  5’-TAAGCCTCCGACTTGTGAAGTGGT-3’ |
| GAPDH | 5’-AGTTAGCCGCATCTTCTTTTGC-3’  5’-CAATACGACCAAATCCGTTGACT-3’ |
